# A protein-coding gene expression atlas from the brain of pregnant and non-pregnant goats

**DOI:** 10.3389/fgene.2023.1114749

**Published:** 2023-07-14

**Authors:** María Gracia Luigi-Sierra, Dailu Guan, Manel López-Béjar, Encarna Casas, Sergi Olvera-Maneu, Jaume Gardela, María Jesús Palomo, Uchebuchi Ike Osuagwuh, Uchechi Linda Ohaneje, Emilio Mármol-Sánchez, Marcel Amills

**Affiliations:** ^1^ Centre for Research in Agricultural Genomics (CRAG), CSIC-IRTA-UAB-UB, Bellaterra, Spain; ^2^ Department of Animal Health and Anatomy, Universitat Autònoma de Barcelona, Bellaterra, Spain; ^3^ Department of Animal Medicine and Surgery, Universitat Autònoma de Barcelona, Bellaterra, Spain; ^4^ Departament de Ciència Animal i dels Aliments, Universitat Autònoma de Barcelona, Bellaterra, Spain

**Keywords:** goat, RNA-Seq, gestation, embryonic vesicle, differential gene expression (DGE), encephalon

## Abstract

**Background:** The brain is an extraordinarily complex organ with multiple anatomical structures involved in highly specialized functions related with behavior and physiological homeostasis. Our goal was to build an atlas of protein-coding gene expression in the goat brain by sequencing the transcriptomes of 12 brain regions in seven female Murciano-Granadina goats, from which three of them were 1-month pregnant.

**Results:** Between 14,889 (cerebellar hemisphere) and 15,592 (pineal gland) protein-coding genes were expressed in goat brain regions, and most of them displayed ubiquitous or broad patterns of expression across tissues. Principal component analysis and hierarchical clustering based on the patterns of mRNA expression revealed that samples from certain brain regions tend to group according to their position in the anterior-posterior axis of the neural tube, i.e., hindbrain (pons and medulla oblongata), midbrain (rostral colliculus) and forebrain (frontal neocortex, olfactory bulb, hypothalamus, and hippocampus). Exceptions to this observation were cerebellum and glandular tissues (pineal gland and hypophysis), which showed highly divergent mRNA expression profiles. Differential expression analysis between pregnant and non-pregnant goats revealed moderate changes of mRNA expression in the frontal neocortex, hippocampus, adenohypophysis and pons, and very dramatic changes in the olfactory bulb. Many genes showing differential expression in this organ are related to olfactory function and behavior in humans.

**Conclusion:** With the exception of cerebellum and glandular tissues, there is a relationship between the cellular origin of sampled regions along the anterior-posterior axis of the neural tube and their mRNA expression patterns in the goat adult brain. Gestation induces substantial changes in the mRNA expression of the olfactory bulb, a finding consistent with the key role of this anatomical structure on the development of maternal behavior.

## 1 Introduction

The mammalian brain is an extraordinarily complex organ integrating multiple highly specialized structures involved in the regulation of memory, behavior, learning, sensory function, motor skills and body homeostasis, amongst others. During embryo development, three vesicles emerge from the neural tube (Chhetri and Das, 2021), which roughly correspond to the forebrain (prosencephalon), midbrain (mesencephalon) and hindbrain (rhombencephalon). Further subdivisions take place in the forebrain (telencephalon and diencephalon) and hindbrain (metencephalon and myelencephalon), resulting in the formation of five encephalic vesicles (Chhetri and Das, 2021). In the bovine embryo, these five encephalic vesicles are visible at 24 days after conception, but they do not become fully developed until 110 days of gestation (Ferreira et al., 2018). Multiple anatomical structures with specialized functions (Chhetri and Das, 2021) are subsequently derived from the telencephalon (cerebral hemisphere, basal ganglia, hippocampus, olfactory bulb, lateral ventricles, etc.), diencephalon (thalamus, hypothalamus, pineal body, neurohypophysis, infundibulum, third ventricle, etc.), mesencephalon (rostral colliculus, tegmentum, crus cerebri, cerebral aqueduct, etc.), metencephalon (pons, cerebellum, upper part of the fourth ventricle, etc.) and myelencephalon (medulla oblongata, spinal cord lower part of the fourth ventricle, etc.).

Transcriptomic analyses may hold the key to significantly advance our knowledge about the biological functions of brain regions. In mice, patterns of gene expression have been used to establish a molecular atlas of the adult brain (Ortiz et al., 2020), and as much as 737 brain structures have been identified with microscopy techniques complemented with other approaches, providing a comprehensive view about the high functional complexity of this organ (Erö et al., 2018). An important feature of the brain is its high plasticity, so any atlas of gene expression is necessarily dynamic, not only in space but also in time. For instance, pregnancy in mice is associated with extensive changes in gene expression in the neocortex, cerebellum, hippocampus and hypothalamus, and there is evidence that several of such modifications might be long lasting (Ray et al., 2016; Arbeitman, 2019).

Very few atlases of brain gene expression have been generated in domestic animals. Recently, Sjöstedt et al. (2020) investigated the gene expression profiles of 10 major mammalian brain regions in humans, mice, and pigs. They found that global transcriptomic profiles are, in general, well conserved in these three species, with cerebrum and brainstem regions clustering apart in hierarchical trees, and the cerebellum showing a highly divergent profile of gene expression (Sjöstedt et al., 2020). The mRNA expression of several brain regions has also been reported in sheep (Clark et al., 2017) and cattle (Harhay et al., 2010), although not comprehensively or systematically. In goats, mRNA expression of two neural tissues, frontal lobe cortex and cerebellum, has also been characterized by RNA-Seq (Muriuki et al., 2019). The main goal of the current work was to establish an atlas of protein-coding gene expression of the caprine brain by sequencing the transcriptomes of 12 encephalic regions in seven Murciano-Granadina goats. Given that three of these goats were pregnant at the time of slaughter, we have also investigated whether gestation affects the transcriptomic profiles of the 12 brain regions under study.

## 2 Materials and methods

### 2.1 Sample collection

Seven adult non-lactating Murciano-Granadina goats raised in the experimental farm of the Faculty of Veterinary Sciences at the Universitat Autònoma de Barcelona (UAB) were slaughtered because of reasons unrelated with this project. Sampled goats were kept under the same management and environmental conditions and had similar ages (6.28 ± 1.38 years). Besides, three of these goats were 1-month pregnant at the time of slaughtering. To minimize pain, goats were administered pentobarbital (150 mg/kg) in the jugular vein. A lethal intravenous dose of sodium pentobarbital causes a mammal to lose consciousness within seconds and results in clinical death within just minutes, but we cannot completely rule out the possibility that the euthanasia procedure used in the current work might have altered the brain mRNA expression of certain genes. Since animals were killed due to the routine culling process implemented in this experimental farm, no permission from the Ethics Committee on Animal and Human Experimentation at UAB was required.

After slaughtering, goats were transported to the Necropsy room of the Faculty of Veterinary Sciences at UAB. The cranial vault of each goat was opened with a bone saw and twelve brain anatomical structures were carefully dissected and biopsied by an expert anatomist. Samples were drawn from the adenohypophysis, cerebellar hemisphere, cerebellar trunk or peduncle, frontal neocortex, hippocampus, hypothalamus, medulla oblongata, neurohypophysis, pineal gland, pons, olfactory bulb and rostral colliculus. Biopsies were immediately submerged in RNAlater (Thermofisher Scientific, Barcelona, Spain) to be stored at -80°C until processing.

To purify total RNA, tissue samples were mixed with 1 ml QIAzol (QIAGEN Inc., Barcelona, Spain) and homogenized using the Lysing Matrix D reagent (MP Biomedicals, Santa Ana, CA) in a Precellys 24 tissue homogenizer (Bertin Instruments, Rockville, MD). The extraction of total RNA was performed using the RNeasy lipid tissue mini kit (QIAGEN Inc., Barcelona, Spain) following the protocol described by the manufacturer. The concentration and purity of extracted RNA molecules were analyzed using the Nanodrop ND-1000 spectrophotometer (Thermofisher Scientific, Barcelona, Spain) and RNA integrity was assessed with a Bioanalyzer-2100 equipment (Agilent Technologies, Santa Clara, CA) using the RNA 6000 Nano Kit 4.2 (Agilent Technologies, Santa Clara, CA).

### 2.2 Sequencing of total RNA

Paired-end sequencing (2 x 50 bp) of total RNA was carried out at the Centre Nacional de Anàlisi Genòmica (CNAG). Sequencing methods have been reported by Guan et al. (2020). Briefly, the RNA-Seq library was prepared with the KAPA Stranded mRNA-Seq Illumina Platforms Kit (Roche, Sant Cugat, Spain) by using 500 ng total RNA as template. Oligo-dT magnetic beads were used to enrich the poly-A fraction and subsequently, RNA was fragmented. Strand cDNA synthesis was performed in the presence of dUTP to enforce strand-specificity. The blunt-ended double stranded cDNA was 3′-adenylated and ligated to Illumina adaptors with unique dual indexes and unique molecular identifiers (Integrated DNA Technologies, Coralville, IA). Enrichment of the ligation product was ensured by performing 15 cycles of polymerase chain reaction amplification. An Agilent 2100 Bioanalyzer equipment was employed to verify the quality of the final library by using the DNA 7500 assay (Agilent Technologies, Inc., Santa Clara, CA). Library sequencing was carried out with a HiSeq 4000 equipment (Illumina, San Diego, CA) in accordance with the protocol for dual indexing advised by the manufacturer. Image analysis, base calling and quality scoring of the sequencing run were checked with the Real-Time Analysis (RTA 2.7.7) tool (Illumina, San Diego, CA) and FASTQ sequence files were subsequently generated.

### 2.3 Quality control, alignment and quantification

The quality of the sequences was assessed with the FastQC software v.0.11.9 (http://www.bioinformatics.babraham.ac.uk/projects/fastqc/) and adapters were trimmed using the TrimGalore v.0.6.6 tool (https://www.bioinformatics.babraham.ac.uk/projects/trim_galore/). In addition, reads with more than five ambiguous bases (Ns) were removed. Filtered sequences were aligned to the goat ARS1 reference genome (Bickhart et al., 2017) with the HISAT2 v.2.2.1 aligner using paired end option (-1 and -2) and default parameters (Kim et al., 2015). Gene assembly and expression quantification were performed with StringTie v.2.1.0 by extracting, with the -e option, those genes annotated in the reference GTF file as reported in a previous protocol (Pertea et al., 2016). The estimated count matrix was obtained from coverage values of each feature (genes in our case) using the dedicated script “prepDE.py” from the StringTie pipeline and based on the following formula (https://github.com/gpertea/stringtie/blob/master/prepDE.py):

$$Reads\;per\;gene=\frac{coverage\times gene\;length}{read\;length}$$

### 2.4 Construction of a brain atlas based on the expression of protein-coding genes

Genes expressed in each tissue were cataloged according to their Ensembl biotype. To remove systematic technical effects (e.g., library size), count data from protein-coding genes were normalized to CPM with the edgeR package (Robinson et al., 2009). Finally, data were log_2_ transformed adding a pseudo-count of 1. Each tissue was normalized separately. Normalization factors were estimated by jointly considering pregnant and non-pregnant goats because library size features were quite similar in both groups. To perform downstream analyses, only genes with a CPM over 0.5 in at least two samples were considered.

In order to detect errors during the sampling procedure, which might alter our results (accidental cross-contaminations when retrieving samples, errors when annotating the anatomical origin of samples etc), the quality of the regional sampling of the brain was evaluated in two ways. First, Euclidean distances were estimated in a pairwise manner based on the corrected level of gene expression (log_2_(CPM + 1)) considering all protein-coding genes (17,054 genes) to identify outlier samples. The distribution of distances between samples from the same tissue was analyzed using the boxplot.stats function from the grDevices package in R (https://r-universe.dev/manuals/grDevices.html), and samples deviating more than 1.5 times from the interquartile range were considered as outliers and, in consequence, eliminated. Additionally, we built a heatmap based on the gene expression level of 61 genes selected by their high tissue-specificity in a set of 12 human tissues (https://www.proteinatlas.org/, version 22) equivalent to those investigated in goats herewith. This analysis was performed to make sure that the expression patterns of the goat samples match what is expected based on human transcriptomic data.

A PCA was constructed by using the *prcomp* function from the stats R package (R Core Team, 2015). Additionally, a hierarchical clustering dendrogram was built based on Euclidean distances and applying the Ward D clustering method with the *hclust* function from the R stats package (Murtagh and Legendre, 2014). For each brain region, the top 1,000 genes with the highest expression levels were selected to perform pathway enrichment analyses with the Enrichr R package (Chen et al., 2013). The KEGG release 99.0 human database (Kanehisa et al., 2016) was used as reference to annotate pathways enriched in the 12 sets of genes (one for each brain region). In order to identify such enriched pathways, a combined score (*c*) was estimated from the output of a traditional Fisher exact test and a Z-score was estimated from expected ranks and variances of the set of genes. In accordance with Chen et al. (2013) the following formula was applied:

$$c=\log\left(p\right)\times z$$

where *c* is the combined score, *p* corresponds to *p*-values estimated with the Fisher exact test, and *z* is the Z-score from the expected rank.

The tissue-specificity expression of protein-coding genes was assessed by calculating the tissue specificity index tau (τ) defined by Yanai et al. (2005):

$$\tau =\frac{\sum_{i=1}^{n}\left(1-x_{i}\right)}{n-1}$$

where *n* is the number of brain regions, and *x*_*i*_ corresponds to the expression profile component normalized by the maximal component value (Yanai et al., 2005). This calculation was performed using the tspex v.0.6.1 program (Camargo et al., 2020). A τ-value of 0 would correspond to a housekeeping gene with ubiquitous tissue expression. In contrast, genes with a restricted regional expression show τ-values above 0.85, while genes with *τ* = 1 can be assumed to be expressed in a single brain region.

### 2.5 Differential expression and pathway enrichment analyses

Before doing the differential expression analysis, the level of variability across replicates from the same condition (pregnant or non-pregnant) was assessed by calculating the coefficient of variation (*CV*) of the expressed genes and genes differentially expressed between conditions:

$$CV=\frac{\sigma }{\mu }$$

where μ is the average expression of each gene and σ is the corresponding standard deviation.

The software DEseq2 (Love et al., 2014) was used to perform a differential expression analysis comparing the transcriptomic profiles of pregnant vs. non-pregnant goats. Genes with a number of estimated counts below 10 were removed. Correction for multiple testing was applied using the false discovery rate (FDR) method (Benjamini and Hochberg, 1995). We considered that a gene is differentially expressed when two conditions are met: 1) absolute logarithm of the fold change |log_2_FC| > 0.58, and 2) Statistical significance threshold: *q-*value < 0.05. Differentially expressed genes were used to perform pathway enrichment analyses using the Enrichr R package (Chen et al., 2013). The non-pregnant goats were considered as the baseline reference, meaning that any upregulation or downregulation of gene expression is referred to changes in the pregnant goats with regard to the non-pregnant. The annotation of differentially expressed genes (DEGs) was performed as explained in section 2.4, while the enrichment pathway analyses were carried out independently in the sets of upregulated and downregulated genes. The level of enrichment was estimated by calculating a *c* score (Chen et al., 2013), as previously defined.

## 3 Results

### 3.1 Sequencing of total RNA from 12 goat brain regions

The average RNA integrity number (RIN) of the 84 RNA extractions was 7.53 ± 0.62, ranging from 6.2 to 9 (Table 1). These values are in agreement with the conservative threshold of RNA degradation (RIN = 6.4–7.9) defined by Gallego Romero et al. (2014) for the in-depth analysis of RNA transcripts. As shown in Table 1, the average sequencing depth ranged from 37.78 to 41.71 million paired-end reads per sample across all 12 brain regions, and the average alignment rate ranged from 84.44% (adenohypophysis) to 94.35% (pons). The read counts per gene per sample, including pregnant and non-pregnant goats are available in Supplementary Table S1.

**TABLE 1:** Quality of RNA samples and number of genes expressed in 12 brain goat regions. Average RNA integrity number (RIN), alignment rate and number of expressed genes (CPM > 0.5 in at least two samples) per brain region in seven Murciano-Granadina female goats.

Brain region	RIN Mean ± SD	Average alignment rate (%)	Average number of reads (millions) Mean ± SD	Number of expressed protein-coding genes (CPM > 0.5)
Adenohypophysis	7.900 ± 0.428	84.44	37.786 ± 2.348	15,220
Cerebellar hemisphere	8.100 ± 0.486	94.11	41.461 ± 4.571	14,889
Cerebellar trunk/peduncle	8.143 ± 0.550	94.17	42.117 ± 9.052	14,898
Frontal neocortex	7.400 ± 0.569	94.26	41.361 ± 7.438	15,098
Hippocampus	7.629 ± 0.411	93.98	41.717 ± 7.825	15,437
Hypothalamus	6.943 ± 0.326	93.92	38.618 ± 4.817	15,557
Medulla oblongata	6.843 ± 0.181	94.27	40.141 ± 5.510	15,420
Neurohypophysis	7.771 ± 0.482	87.8	39.443 ± 5.931	15,366
Olfactory bulb	7.214 ± 0.422	94.16	40.139 ± 3.938	15,581
Pineal gland	8.029 ± 0.594	94.23	39.228 ± 7.142	15,592
Pons	7.186 ± 0.654	94.35	39.505 ± 4.233	15,273
Rostral colliculus	7.243 ± 0.412	93.63	38.832 ± 3.179	15,153

### 3.2 Clustering of brain regions according to their mRNA expression profile

The total number of protein-coding loci expressed (counts per million, CPM > 0.5 in at least two samples) in each tissue ranged from 14,889 genes for the cerebellar hemisphere to 15,592 genes for the pineal gland. Considering all brain regions, 17,054 protein-coding genes passed the quality control (QC) filters and were expressed in at least two samples. One pineal gland sample deviated more than 1.5 times from the interquartile range, so it was identified as an outlier and excluded from any further analysis. As a complementary measure to detect sampling errors, we built a heatmap based on the expression levels of 61 marker genes which in humans show high tissue specificity for each one of the brain regions considered in this research. As shown in Supplementary Figure S1, the expression patterns of the samples based on the selected 61 genes is concordant across replicates from the same brain region, thus evidencing that dissection and sampling were correctly performed. In general, all brain regions display high expression levels of the marker genes in close correspondence to expectations based on human data, with the only exception of pons and medulla oblongata. Samples from the medulla oblongata display high mRNA levels of genes *PHOX2A* and *SLC6A2,* which are considered markers for the pons based on human data. In congruence with our results, the expression of these genes is reported to be high in the medulla oblongata of mice (Sunkin et al., 2013). Furthermore, the gene *EXD1* selected as a marker for pons was highly expressed in the pineal gland of goats. This gene is also expressed at high levels in the pituitary gland of pigs, and at moderate levels in multiple brain structures of pigs and mice (Sjöstedt et al., 2020). We also evaluated the variability of the mRNA expression patterns across replicates by calculating the corresponding coefficient of variation per group. In general, the coefficient of variation was lower within the pregnant and not pregnant groups than when all samples were pooled together in a single group (Supplementary Figure S2). This result was even more evident when analyzing the sets of differentially expressed genes in brain regions moderately or highly affected by pregnancy (Supplementary Figure S2B).

**FIGURE 1:**
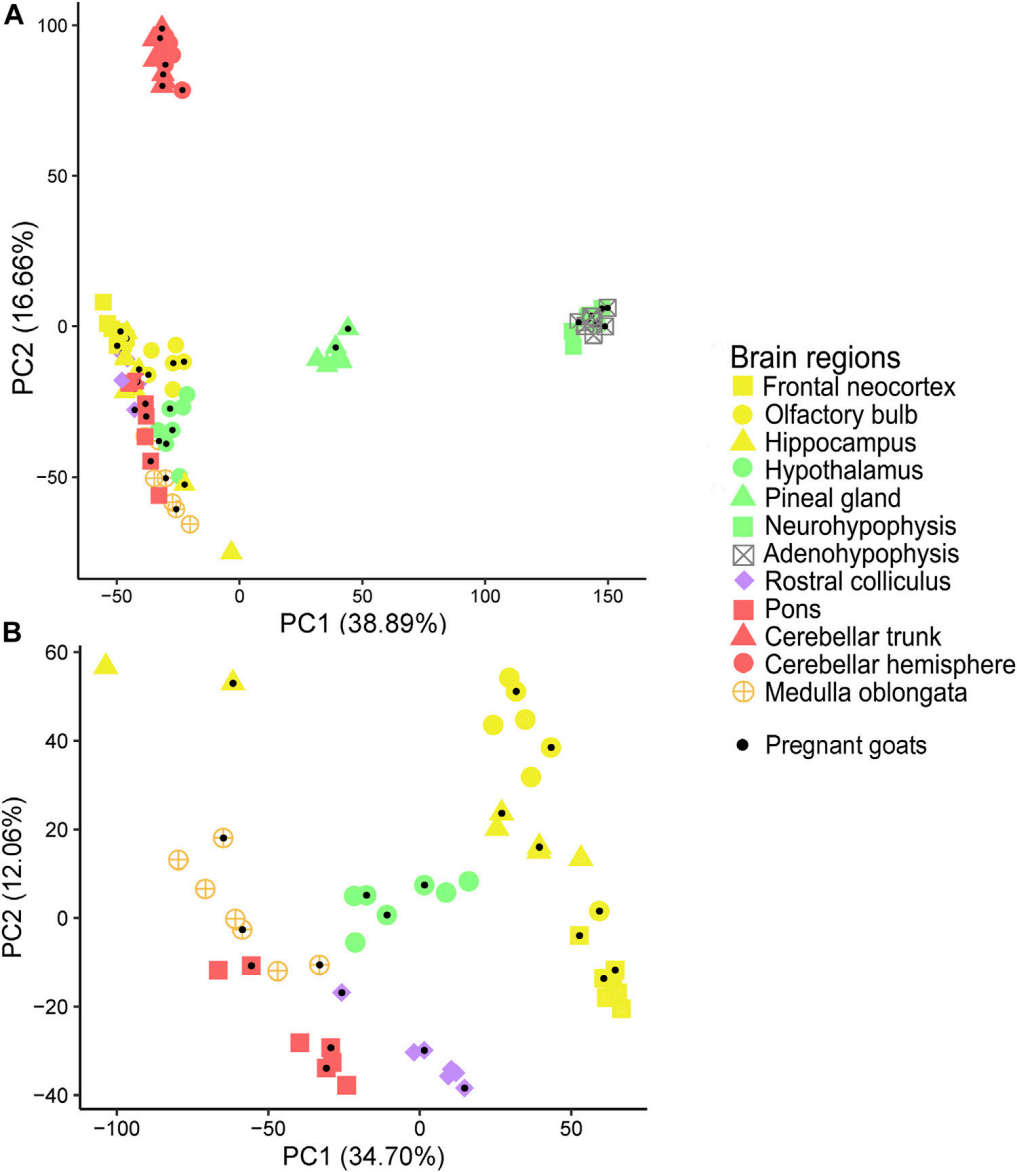
Principal component analysis (PCA) of samples from 12 goat brain regions. **(A)** Principal component analysis (PCA) of samples from 12 brain regions (adenohypophysis, cerebellar hemisphere, cerebellar trunk, frontal neocortex, hippocampus, hypothalamus, medulla oblongata, neurohypophysis, pineal gland, pons, olfactory bulb and rostral colliculus) retrieved from seven Murciano-Granadina female goats. **(B)** Same PCA without samples from adenohypophysis, cerebellar hemisphere, cerebellar trunk/peduncle, neurohypophysis, and pineal gland. Samples obtained from pregnant goats have been marked with a black dot.

**FIGURE 2:**
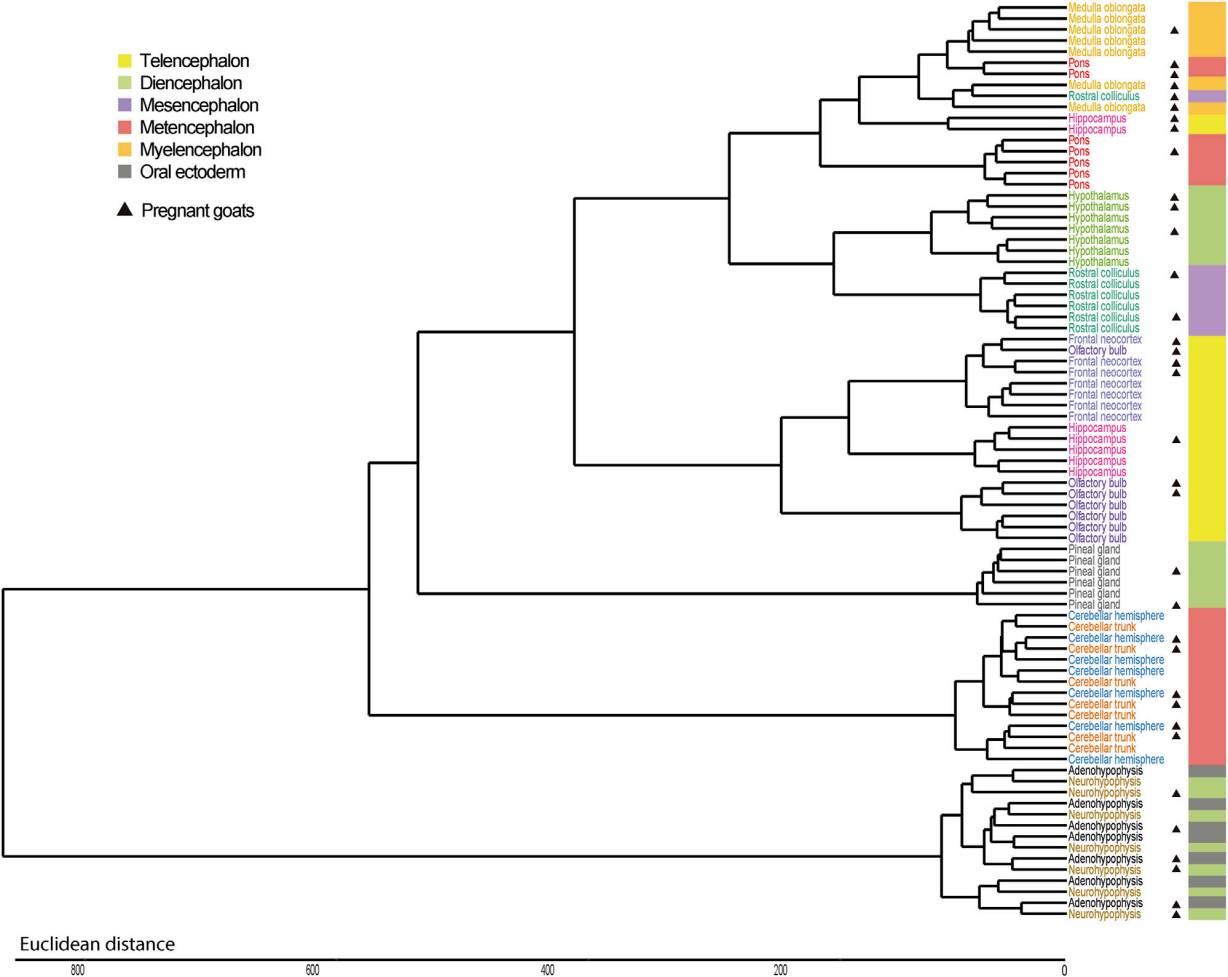
Hierarchical clustering of samples from 12 brain regions retrieved from seven Murciano-Granadina goats based on their mRNA expression profiles. Dendrogram displaying the clustering patterns of samples from 12 brain regions. In most cases, samples cluster according to their region of origin and, moreover, regional clusters tend to group according to the forebrain, midbrain or hindbrain embryonic vesicle they originate from. Exceptions to this general observation are hypothalamus, that groups with rostral colliculus, and two main groups of outliers: cerebellum and glandular tissues (pineal gland and hypophysis). In the right part of the figure, we indicate the embryonic vesicle from which each region is derived. In an initial developmental stage of mammals, three forebrain (prosencephalon), midbrain (mesencephalon) and hindbrain (rhombencephalon) vesicles are formed. Subsequently, prosencephalon is subdivided into two further vesicles (telencephalon and diencephalon) and so does rhombencephalon (metencephalon and myelencephalon). Adenohypophysis does not originate from any of these five vesicles, but from the oral ectoderm. Samples obtained from pregnant goats have been marked with a black triangle on the side.

In close agreement with data reported above, the PCAs, with (Figure 1A) and without (Figure 1B) cerebellum and glandular samples, and the dendrogram (Figure 2) showed that most samples clustered according to their region of origin. We also observed that four regions, namely adenohypophysis, neurohypophysis, pineal gland, and cerebellum (hemisphere and trunk), displayed highly divergent gene expression patterns when compared to the remaining encephalic regions (Figure 1A; Figure 2). The dendrogram indicated that pons and medulla oblongata, which are hindbrain structures, tended to group together. Moreover, frontal neocortex, olfactory bulb, and hippocampus, which derive from the forebrain telencephalon vesicle, also tended to group together. In strong contrast, cerebellum trunk and hemisphere clustered far apart from any other brain region (including pons despite its anatomical proximity), and adenohypophysis (derived from the oral ectoderm) and neurohypophysis (derived from the diencephalon), which have completely different embryonic origins, also grouped jointly and clearly separated from the remaining brain structures.

### 3.3 Regional specificity of mRNA expression

**FIGURE 3:**
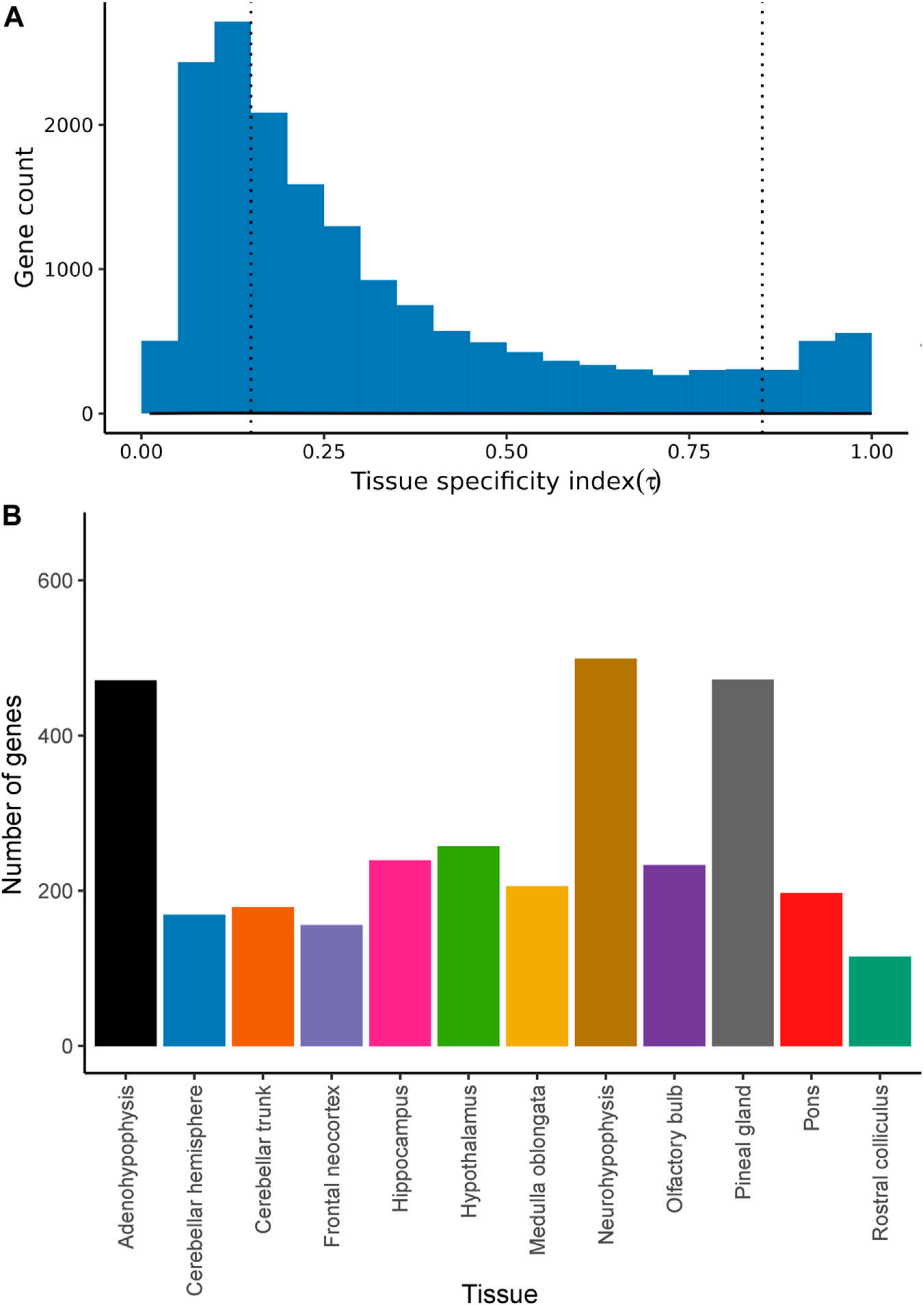
Tissue specificity of protein-coding genes expressed in 12 goat brain regions. **(A)** Histogram representing the tissue specificity of 17,054 protein-coding genes expressed in 12 goat brain regions (in at least one sample) and surpassing QC filters. The number of genes is indicated in the *y*-axis, while Tau specificity scores (τ, see Materials and Methods for details) are shown in the *x*-axis. The dashed line indicates = 0.85 threshold that defines tissue-specificity. **(B)** Number of genes with tissue-specific expression (τ > 0.85) in each of the 12 brain regions under study.

The distribution of the tissue-specificity index τ-values was highly skewed to the left (Figure 3A). Indeed, 5,655 (33.16%) protein-coding genes showed τ-values below 0.15, indicative of a highly ubiquitous pattern of expression across all brain regions (Supplementary Table S2A). In contrast, 10,036 (58.84%) protein-coding genes displayed intermediate τ-values (*τ* = 0.15–0.85) and 1,363 protein-coding genes (7.99%) had a highly region-specific profile (*τ* > 0.85) of mRNA expression (Figures 3A, B). A heatmap based on the level of expression of tissue-specific genes (*τ* ≥ 0.85) identified for each tissue is shown in Supplementary Figure S3.

As shown in Supplementary Table S2B, the gene pathway enrichment analysis for loci with an ubiquitous pattern of expression (*τ* = 0–0.15) revealed that they are mainly involved in general biological processes such as RNA transport (combined score, *c* = 110.230, *q-*value *=* 1.16 E-13), autophagy (*c =* 105.214, *q-*value *=* 5.66 E-12), oxidative phosphorylation (*c* = 110.941, *q-*value *=* 3.34 E-12), spliceosome (*c* = 124.950, *q-*value *=* 1.16 E-13), proteasome (*c* = 185.773, *q-*value *=* 5.52 E-10) and ubiquitin mediated proteolysis (*c* = 127.938, *q-*value *=* 1.56 E-13), as well as in the development of neurological diseases such as amyotrophic lateral sclerosis (*c* = 213.777, *q-*value *=* 5.57 E-26), Parkinson disease (*c* = 195.845, *q-*value *=* 1.86 E-21), and Huntington disease (*c* = 142.384, *q-*value *=* 3.91 E-19), to mention a few.

As indicated in Supplementary Table S3 and Supplementary Figures S4–S15, we have also investigated whether genes with highly region-specific gene expression patterns (*τ* = 0.85–1, listed in Supplementary Table S3A) are enriched in specific functional pathways in all analyzed regions (Supplementary Table S3B–S3M). The neuroactive ligand-receptor interaction pathway was highly enriched in genes with *τ* = 0.85–1 in several of the analyzed regions, i.e., frontal neocortex, hippocampus, hypothalamus, and olfactory bulb. Other pathways associated with neuronal transduction were also enriched in genes with a tissue-restricted expression, e.g., serotonergic synapse (cerebellar hemisphere and cerebellar trunk with *q-*values < 0.05 and *c* of 74.868, and 65.095, respectively) and GABAergic synapse (cerebellar hemisphere and trunk, with *q-*values < 0.05 and *c* of 69.725 and 60.823, respectively). Besides, the cholinergic synapse pathway showed enrichment in the rostral colliculus (*c* = 43.169, *q-*value *=* 0.05). Several brain regions showed an enrichment pattern that agreed well with their biological functions (Supplementary Tables S3B–S3M). For instance, in the pineal gland the phototransduction pathway showed a *c* of 1101.90 (*q-*value *=* 1.06 E-13).

### 3.4 KEGG pathway enrichment analysis of highly expressed genes

The results of the Kyoto encyclopedia of genes and genomes (KEGG) pathway enrichment analysis for the 1,000 mRNA genes with the highest expression in each region (Supplementary Tables S4A–S4K) revealed several pathways broadly shared across brain regions and also reaching strong statistical significance (*q*-value < 0.05), e.g., synaptic vesicle cycle, endocrine and other factor-regulated calcium reabsorption, endocytosis, phagosome, long-term potentiation, dopaminergic synapse, glutamatergic synapse, circadian entrainment, gap junction, gastric acid secretion and adrenergic signaling in cardiomyocytes. We also detected many pathways related to neurological conditions that attained high statistical significance (*q*-value < 0.05) and were represented in a broad array of encephalic regions, e.g., Alzheimer disease, Huntington disease, Parkinson disease, prion disease, amyotrophic lateral sclerosis, and, to a much lesser extent, spinocerebellar ataxia. Pathways related with *Vibrio cholerae* and *Salmonella* infection were also commonly found. Protein processing in endoplasmic reticulum (*q*-value < 0.05), RNA transport (*q*-value < 0.05) and ribosome (*q*-value < 0.05) were amongst the most significant pathways in the adenohypophysis (Supplementary Table S4A) and neurohypophysis (Supplementary Table S4H), but not in the other regions. As said before, in the pineal gland (Supplementary Table S4J) phototransduction was also a highly significant pathway (*q-*value = 0.006) while in the remaining brain regions it did not reach statistical significance.

### 3.5 Analysis of differential gene expression in pregnant and non-pregnant goats

**FIGURE 4:**
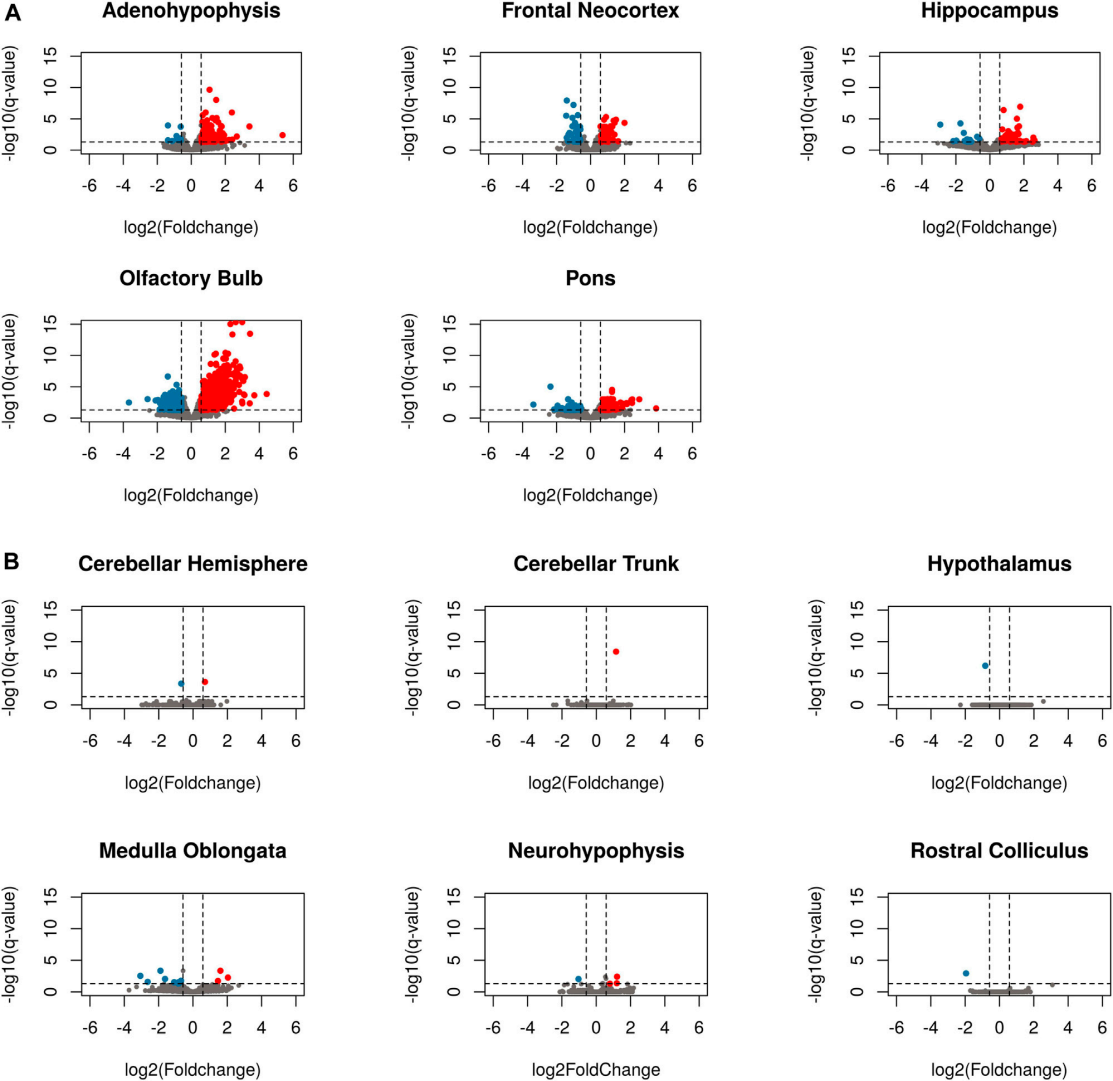
Volcano plots of differentially expressed genes in eleven brain regions when comparing pregnant and non-pregnant goats. **(A)** Brain regions little affected by pregnancy, i.e., cerebellar hemisphere, cerebellar trunk/peduncle, hypothalamus, medulla oblongata, neurohypophysis and rostral colliculus. **(B)** Brain regions moderately or strongly affected by pregnancy, i.e., adenohypophysis, frontal neocortex, hippocampus, olfactory bulb, and pons. Genes with a fold change below -1.5 and a *q-*value below 0.05 are depicted in blue, while genes with a fold change over 1.5 and a *q-*value < 0.05 are depicted in red. Grey dots represent genes that do not display differential expression.

Samples from the pineal gland were not included in the differential expression analysis, since the removal of an outlier sample decreased the number of replicates from the pregnant group to two, which is insufficient to make a sound statistical analysis. When comparing pregnant vs. non-pregnant goats, brain regions showed strong differences in terms of the number of differentially expressed genes. Six regions displayed little changes in their expression levels in response to 1 month-pregnancy: in the cerebellar hemisphere, cerebellar trunk, hypothalamus, medulla oblongata, neurohypophysis and rostral colliculus, only 2, 1, 1, 12, 4 and 1 DEGs were identified, respectively (Figure 4B, Supplementary Table S5). In strong contrast, we observed remarkable changes in the expression profiles of five brain regions (Figure 4A, Supplementary Table S5): adenohypophysis (201 DEGs, 13 downregulated and 188 upregulated in pregnant goats, Supplementary Table S5A), frontal neocortex (82 DEGs, 37 downregulated and 45 upregulated in pregnant goats, Supplementary Table S5D), hippocampus (70 DEGs, 15 downregulated and 55 upregulated in pregnant goats, Supplementary Table S5E), pons (190 DEGs, 57 downregulated and 133 upregulated in pregnant goats, Supplementary Table S5J) and, most remarkably, olfactory bulb (1207 DEGs, 381 downregulated and 826 upregulated in pregnant goats, Supplementary Table S5I). Pathways significantly associated with DEGs in each tissue are shown in Supplementary Tables S6A–S6E and Supplementary Figures S16–S20. In the olfactory bulb (Supplementary Table S6D), the majority of biological functions enriched in the set of upregulated DEGs in pregnant goats were associated with axon guidance (*c* = 89.246; *q-*value *=* 7.29 E-07), dopaminergic synapse (*c* = 79.526; *q-*value *=* 6.07 E-06), neuroactive ligand-receptor interaction (*c* = 53.217; *q-*value *=* 5.27 E-06), cholinergic synapse (*c* = 55.712; *q-*value *=* 1.32 E-04) and phenylalanine, tyrosine and tryptophan biosynthesis (*c* = 53.087; *q-*value *=* 8.51 E-02). In contrast, downregulated DEGs in the olfactory bulb of pregnant goats were enriched in pathways related to nervous development and control of cellular activities, including migration, differentiation, and proliferation (Supplementary Table S6D). A heatmap based on the expression levels of genes differentially expressed in five tissues moderately or highly affected by pregnancy (i.e., adenohypophysis, frontal neocortex, hippocampus, olfactory bulb, and pons) is shown in Supplementary Figure S21. It can be seen that the main determinant of gene expression patterns is the region-of-origin of samples rather than pregnancy.

By making a literature search for each one of the genes showing differential expression in the olfactory bulb of pregnant vs. non-pregnant goats, we observed that many of them are related to human behavioral traits (Supplementary Table S7), including, but not limited to: maternal behavior (e.g., downregulated: *DDC*; upregulated: *DBH, DRD1, NTS* and *HTR2A*), affective behavior, sociability and exploration (e.g., downregulated: *FGFR4, GRIP2, NTRK2* and *RYR3*; upregulated: *HTR2C, FBX O 45, GPR3, KCNQ2* and *PLXNA2*), anxiety and depression (e.g., downregulated: *NOS2, PAN2* and *H3-3B*; upregulated: *BSCL2, HRH1, SIK2, PDYN, GLRB* and *NRN1*), autism (e.g., downregulated: *MOCOS, CACNA1D, MBD6* and *AUTS2*; upregulated: *RAB39B, BTBD11, KCNQ3, CDH9, CADPS2, DOCK4* and *EXT1*), aggression (e.g., downregulated: *NOS1*; upregulated: *HRH3, PRNP, HNMT* and *GRIA3*), cognition, memory and learning (e.g., downregulated: *EPHA10, STAT5* and *MMP28*; upregulated: *EPHA6, SYP, SORBS2, ARHGEF4, HCN1, CAMK2N2, MMP17, BTBD9, CLSTN3, STAU2RIMKLA, PAK6, SLC22A4* and *NEURL1*), response to stress (e.g., downregulated: *IFIT1*; upregulated: *PDYN, EPOP, DPYSL2* and *HCN2*), feeding behavior (e.g., upregulated: *NELL2, CXCL14, GPR45, GPR162, NPY* and *ACBD7*) and diverse neuropsychiatric disorders (e.g., downregulated: *SLITRK6, IL1RAPL1, BAHCC1, WDR62* and *SLC6A1*; upregulated: *SCN1A, GABBR2, RTN4R, ACOT7, KIF5A. HECW2, NRG1, SNCA, ATP8A2, WFS1, HTR5A, STX1A* and *JPH3*).

As shown in Supplementary Table S8, our literature search also revealed several DEGs associated with the migration and maturation of olfactory bulb interneurons (e.g., *PROKR2* and *SALL3*), olfactory bulb angiogenesis (e.g., *ANOS1*), olfactory bulb morphogenesis and establishment of a functional olfactory neural circuitry (e.g., *ARX, TSHZ1, OLFM2* and *FGFR1*), axonal growth and guidance (e.g., *RAP1GAP2, SHH, GLI3* and *DSCAM*) and sensitivity of olfactory sensory neurons to food cues (e.g., *NPY*). A heatmap displaying the gene expression patterns of 207 genes related to behavior (Supplementary Table S7) and 53 genes involved in olfaction (Supplementary Table S8) that show differential expression in the olfactory bulb of pregnant vs. non-pregnant goats is shown in Supplementary Figure S22.

## 4 Discussion

### 4.1 The clustering of samples from certain brain regions follows an anterior-posterior pattern consistent with embryonic origin

The majority of the 17,054 protein-coding genes expressed in the goat brain had τ-values below 0.25 (55.57% of the genes), implying that they have a ubiquitous pattern of expression. Indeed, only 469 genes (2.75%) were expressed in just one of the 12 sampled brain regions (*τ* = 1). Results obtained in the Genotype-Tissue Expression (GTEx) project were consistent with this finding (Melé et al., 2015), since only 200 genes showed tissue-specific expression (95% of these genes were exclusively expressed in the testis).

Brain region of origin, rather than pregnancy, was the major factor explaining the clustering of samples. By using microarrays, Ferraz et al. (2008) investigated the profiles of expression of 16 porcine tissues and observed that the factor “tissue of origin” accounted for ∼11 times more variability than sex or breed. In humans, the GTEx Consortium retrieved 1641 post-mortem samples covering 54 body sites from 175 individuals and reported that tissue type was the primary factor explaining differences in gene expression (Ardlie et al., 2015), a result that is fully consistent with ours. Similar findings have been obtained when building atlases of gene expression in cattle (Harhay et al., 2010), pigs (Freeman et al., 2012) and sheep (Clark et al., 2017). We have observed that a few samples do not cluster with the remaining samples from the same region of origin (see for instance the two hippocampus samples in the upper left quadrant of the PCA shown in Figure 1B). This lack of consistent clustering has been observed in other atlases of gene expression (Harhay et al., 2010; Ardlie et al., 2015; Clark et al., 2017). Rather than mix-ups or contaminations, unexpected patterns of clustering could be due to the expression of thousands of housekeeping genes, that are transcribed at similar rates in different tissues, and, additionally, to the existence of “communication pathways” between tissues which might contribute to generate a high correlation of expression profiles between them, as evidenced in previous studies (Wang et al., 2019).

We have observed that, in goats, frontal neocortex, olfactory bulb and hippocampus samples tend to group together, and the same trend is observed for rostral colliculus, medulla oblongata and pons samples, while hypothalamus samples would be placed in the middle of these two clusters. In strong contrast, cerebellum, pineal gland and adeno/neurohypophysis show highly differentiated patterns of gene expression (Figure 1A; Figure 2). It could be argued that, with few exceptions, brain samples cluster together because of anatomical proximity. However, it should be noticed that the olfactory bulb is located closer to hypothalamus than to the hippocampus, or that the distance between the rostral colliculus and the hippocampus is shorter than that between the olfactory bulb and the hippocampus. The common denominator between frontal neocortex, olfactory bulb and hippocampus is that these three organs derive from the telencephalon, while pons and medulla oblongata come from the rhombencephalon. In the comprehensive analysis of porcine tissue expression carried out by Ferraz et al. (2008), tissues clustered according to the germ layer (ectoderm, mesoderm, or endoderm) they derive from. Measurement of transcriptome profiles in 24 murine neural tissues also highlighted the existence of a relationship between the cellular position along the anterior-posterior axis of the neural tube and gene expression in different regions from the adult brain (Zapala et al., 2005), as also published by Ortiz et al. (2020). These published results and those obtained by us agree with the hypothesis that embryogenesis might leave a durable footprint in the profile of mRNA expression of mammalian brain regions.

### 4.2 Evolutionary conservation of the genome-wide patterns of expression across brain regions

Sjöstedt et al. (2020) described the patterns of genome-wide expression of protein-coding genes in a number of brain regions from humans, mice and pigs. They found that, in general, transcriptomic patterns are evolutionarily conserved in these three species, with the three forebrain structures (cerebral cortex, hippocampus, and amygdala) grouping together, and the midbrain, thalamus, and pons and medulla samples forming another cluster close to the hypothalamus (Sjöstedt et al., 2020). They also found that the cerebellum behaves as an outlier, a finding fully coherent with ours.

Although transcriptomic profiles of brain regions seem to be well conserved across species, relevant differences also exist. For instance, Sjöstedt et al. (2020) indicated that in humans the olfactory bulb clusters with other forebrain structures (cerebral cortex, hippocampus, amygdala, etc.), while in pigs and mice this brain structure almost behaves as an outlier. They reasoned that this might be due to the fact that these two latter species have more evolved olfactory systems than humans (Sjöstedt et al., 2020). In goats, the olfactory bulb is also much more developed than in humans (Kavoi and Jameela, 2011), a feature consistent with the fundamental role of the olfactory system in the maternal and social behaviors of ungulates (Keller and Lévy, 2012). However, our data indicate that in goats the pattern of mRNA expression of the olfactory bulb is closely aligned with that of other forebrain regions (Figure 1B; Figure 2). This result agrees much more with the PCA reported by Sjöstedt et al. (2020) for human brain structures than for those corresponding to pigs or mice. A higher resemblance of goats to humans, rather than to pigs, is unexpected because goats and pigs are ungulates (Benton et al., 2015). Sjöstedt et al. (2020) stated that the functional importance of olfaction might be a key factor explaining the differential patterns of olfactory bulb mRNA expression observed in pigs/mice vs. humans. Such hypothesis is not fully consistent with our results and should be interpreted cautiously until further data are available in other mammalian species.

### 4.3 Cerebellum, pineal gland and hypophysis have highly differentiated patterns of gene expression

Cerebellum displayed a highly differentiated pattern of mRNA expression when compared to other brain structures (Figure 1A; Figure 2), a finding that, as mentioned before, is consistent with previous reports (Ortiz et al., 2020; Sjöstedt et al., 2020). Moreover, cerebellum trunk and hemisphere mRNA profiles were quite similar. Cerebellum is strongly specialized in the learning and coordination of motor activities as well as in the triggering of reflex responses, and it has been suggested that it is scarcely connected with cognitive areas of the brain cortex (Glickstein and Doron, 2008). One of the most distinctive features of the cerebellum is its extraordinarily high cell density: while this organ represents 10% of brain volume, it encompasses 42% of all brain cells and 59.8% of all excitatory neurons, mainly due to the conspicuous abundance of tightly packed granular cells (Erö et al., 2018). Another relevant feature of the cerebellum is that the same circuit, composed by mossy fibers that excite granule cells that, in turn, excite Purkinje cells, forms a fundamental unit that is replicated thousands of times (Kozareva et al., 2020). These biological particularities might have contributed to transform the cerebellum into an organ with a very specific transcriptomic profile, not only in goats but also in other mammals.

We also observed highly divergent patterns of gene expression in three glandular tissues: the pineal gland and the adeno/neurohypophysis (Figure 1A), a finding consistent with results reported by Harhay et al. (2010). The strong functional specialization of these three anatomical structures in hormonal secretion might explain these findings. In response to light, the pineal gland, which develops from an evagination of neuroepithelium in the dorsal midline of the diencephalon, synthesizes and releases melatonin, which is a key regulator of the circadian sleep-wake cycle and seasonal rhythms (Patel et al., 2020). Remarkably, in our study the pineal gland was the second organ showing the highest number of genes with region-specific expression (*τ* > 0.85, Figure 3B and Supplementary Table S3A). Neurohypophysis and adenohypophysis are also specialized in the secretion of molecules with key physiological roles, and data collected in sheep and cattle indicate that their profiles of mRNA expression are highly differentiated from those of other brain structures (Harhay et al., 2010; Clark et al., 2017). The main hormones produced by the adenohypophysis are prolactin, adrenocorticotropic hormone, luteinizing hormone, follicle-stimulating hormone, growth hormone and thyroid-stimulating hormone, which regulate a very diverse set of biological processes including growth, metabolism, lactation, stress and reproduction (Le Tissier et al., 2012). One particular feature of the adenohypophysis is that it does not develop from any of the five neural vesicles but from the oral ectoderm (Le Tissier et al., 2012). In contrast, the neurohypophysis has a diencephalic origin and stores vasopressin and oxytocin (both are synthesized in the hypothalamus), two hormones regulating diuresis and a broad array of reproduction and behavioral processes, respectively (Santos Fontanez and De Jesus, 2021). Despite having completely different embryological origins, histological structure and biological functions, our data indicate that neurohypophysis and adenohypophysis share similar profiles of mRNA expression (Figure 1A; Figure 2). Our interpretation is that high functional specialization in hormonal secretion might erase, at least partially, the transcriptomic footprint associated with embryogenesis, a hypothesis supported by the distinctive patterns of gene expression observed in both pituitary structures when compared with the remainder encephalic regions. Further studies are needed to assess whether such interpretation is correct.

### 4.4 Heterogeneous effects of pregnancy on the expression profiles of eleven goat brain regions

Amongst the seven individuals sampled in our study, there were three pregnant goats providing the opportunity to investigate the effect of pregnancy on the brain transcriptome. Since the number of replicates for each pregnant (N = 3) and non-pregnant (N = 4) category is low, sensitivity to detect DEGs is expected to be quite limited (Schurch et al., 2016). To circumvent this difficulty, we have used the total number of DEGs as the main criterion to identify which brain regions are mostly affected by 1-month pregnancy. In principle, low number of replicates should affect the number of detected DEGs to a similar extent in all 11 brain regions (pineal gland was excluded from the differential expression analysis), so the number of DEGs seems to be an appropriate indicator of which regions are more affected by pregnancy.

In comparisons involving cerebellar hemisphere, cerebellar trunk, hypothalamus, medulla oblongata, neurohypophysis and rostral colliculus, the total number of DEGs when comparing pregnant vs. non-pregnant goats was very low or inexistent (Figure 4B). In contrast, between 70 and 201 DEGs were detected in the frontal neocortex, hippocampus, adenohypophysis and pons (Figure 4A). By far, the organ which displayed the largest number of DEGs was the olfactory bulb (826 upregulated and 381 downregulated genes in pregnant goats). From these data, we conclude that 1-month pregnancy does not have the same effect on all goat brain regions. Ray et al. (2016) investigated changes in the mRNA expression of four brain structures (hypothalamus, neocortex, hippocampus and cerebellum) in virgin, pregnant, and postpartum mice, and they found that in the virgin vs. pregnant comparison the number of DEGs was much higher in the hippocampus than in the cerebellum, while hypothalamus and neocortex showed intermediate values. These results support the notion that pregnancy does not affect all brain regions to the same extent.

Brain gene expression during pregnancy is dynamic and it might change depending on the time point under consideration. By using magnetic resonance imaging, it has been shown that the brain of primiparous women experiences substantial morphological changes during gestation which mostly affect the right middle temporal gyrus, inferior frontal gyrus and posterior cingulate cortex (Hoekzema et al., 2017). A longitudinal morphometric study in mice also provided evidence of transient hypertrophy associated with gestation and/or lactation in the medial preoptic area, bed nucleus of the stria terminalis, amygdala, caudate nucleus, and hippocampus (Barrière et al., 2021). These findings indicate that certain brain structures undergo significant alterations in their profiles of expression and morphology because of gestation, and that biological changes are highly dynamic. Thus, the lists of DEGs detected for 11 goat brain regions at 1-month of gestation might not be representative of the whole gestation, but just of this specific time point.

In the goat frontal neocortex and the hippocampus, 82 DEGs (45 upregulated and 37 downregulated in pregnant goats) and 70 DEGs (55 upregulated and 15 downregulated in pregnant goats) were detected, respectively (Supplementary Tables S5D and S5E). The hippocampus and the frontal neocortex play important roles in modulating memory and learning as well as social behavior (Zemla and Basu, 2017; Reinert et al., 2021), and there is evidence that hippocampal neurogenesis is affected by pregnancy (Rolls et al., 2008). In this way, genes included in pathways involved in the immune response, including *NFATC3* and *CCL2*, and apoptosis (*CTSH*) were upregulated in these tissues in pregnant goats (Supplementary Table S6C). Interestingly, these genes have been associated with neurodegenerative processes in the hippocampus and memory loss (Abdul et al., 2009; Wang et al., 2010). Besides, hippocampal lesion in the female rat results in deficient nest construction and reduced pup survival, suggesting that this organ has a key influence on the development of maternal behavior (Murphy and Wildeman, 1986).

In the adenohypophysis 201 DEGs (188 upregulated and 13 downregulated in pregnant goats) were identified (Supplementary Table S5A). Adenohypophysis provides hormonal signals that are fundamental for the maintenance of gestation. As listed in Supplementary Table S6A, genes included in hormonal pathways like oxytocin signaling pathway (i.e., *RYR2, ROCK2, GNAQ, NFATC3* and *ADCY7*) and cortisol synthesis and secretion (i.e., *NCEH1, GNAQ, CREB3L2* and *ADCY7*) were upregulated in pregnant goats. The anterior pituitary is strongly enlarged during gestation in response to hormones produced by the placenta and ovaries, and the secretion of prolactin increases gradually to prepare the female for lactation (Carlson, 2011).

Differential mRNA expression in the pons, with 190 DEGs (133 upregulated and 57 downregulated in pregnant goats) is harder to interpret (Supplementary Table S5J). This brain region is mostly involved in the regulation of breathing and sleep, and in relaying information to or from the cerebellum to other brain regions (Venkatraman et al., 2017). However, there is evidence that the pons has a basic role in the generation and experience of emotions through the integration of arousal, autonomic function, motor control, and somatosensory signals (Venkatraman et al., 2017). Moreover, it has been reported that preoptic area projections to lower brainstem regions affect maternal behavior in postpartum rats (Numan and Numan, 1991).

### 4.5 Several genes are differentially expressed in two or more brain regions in pregnant vs. non-pregnant goats

We have detected 94 genes that are differentially expressed in more than one tissue when comparing pregnant and non-pregnant goats (90 genes are differentially expressed in two tissues and four genes are differentially expressed in three tissues). The most significant pathways detected in this list of 94 genes are Wnt signalling pathway, adherens junctions signalling pathways, and FoxO signalling pathway (Supplementary Table S9). These pathways are related to cellular growth and proliferation and they are involved in the formation and modulation of neuronal circuits, neurogenesis, neuronal migration and axon guidance (Inestrosa and Varela-Nallar, 2015; Renault et al., 2009; Stocker and Chenn, 2015).

Amongst this list of 94 loci, one gene that is particularly interesting is that encoding the dopa decarboxylase (*DDC*) mRNA, which was consistently downregulated in the pons and olfactory bulb (log_2_FC -2.08 and -1.3, respectively). This gene is responsible for the synthesis of dopamine, essential for a healthy pregnancy (Gratz et al., 2018). Dopamine is a key modulator of maternal behavior in rodents, partly because it inhibits the pituitary release of prolactin which modulates lactation, corpora lutea function (in certain species), and maternal behavior (Nevard et al., 2022).

Notably, the direction of differential gene expression was not always consistent across tissues. For instance, the vesicular associated membrane protein 1 (*VAMP1*) gene was significantly upregulated in the olfactory bulb of pregnant goats (log_2_FC = 1.15), while in the frontal neocortex was downregulated in pregnant goats (log_2_FC = -0.67). The *VAMP1* gene encodes a protein that plays an important role in synapsis and synapsis plasticity and forms part of the soluble N-ethylmaleimide-sensitive factor attachment protein receptor complex (Dalmaz et al., 2021). These proteins are involved in neurocognitive processes and associated with the Wnt pathways that, among other things, are important for neurogenesis and cell proliferation (Dalmaz et al., 2021).

### 4.6 Dramatic changes in the mRNA expression of the olfactory bulb in response to early gestation

The olfactory bulb was the brain region that displayed, by far, the highest number of DEGs (Supplementary Table S5I), implying that this organ is strongly affected by 1 month-pregnancy. The olfactory bulb processes smell information transmitted from olfactory sensory neurons, which express odorant receptors in the olfactory epithelium, and relays it to the olfactory cortex (Nagayama et al., 2014). Importantly, the sense of olfaction is strongly connected to a broad array of behavioral responses related with aversion to food, avoidance of predators, sex arousal, partner preference, and aggression, amongst others (Sullivan et al., 2015). For instance, bilateral bulbectomy of male rats is associated with indifference to the sexual status of females and suppression of mating (Edwards et al., 1990), and it also decreases aggressive behavior between males (Rowe and Edwards, 1971).

Of course, the strong changes in the mRNA expression profile of the olfactory bulb observed in pregnant goats, when compared to their non-pregnant counterparts, are not triggered by odorant signals delivered by the offspring. More likely, this remarkable change of gene expression might be promoted by other brain regions and/or endocrine glands delivering chemical signals to the olfactory bulb. As expected, several DEGs are related with the sense of olfaction (Supplementary Table S8). For instance, the inactivation of the zinc finger homeodomain factor teashirt zinc finger family member 1 (*TSHZ1*, |log_2_FC| 1.36, *q-*value < 0.05) or of the prokineticin receptor 2 (*PROKR2*, |log_2_FC| 1.28, *q-*value < 0.05) genes results in olfactory bulb hypoplasia (Prosser et al., 2007; Ragancokova et al., 2014); and the loss of the Spalt like transcription factor 3 (*SALL3*, |log_2_FC| 1.02, *q-*value < 0.05) inhibits the formation of the glomerular layer in the olfactory bulb (Harrison et al., 2008). The aristaless related homeobox (*ARX*, |log_2_FC| 0.97, *q-*value < 0.05) gene is fundamental for the proper development of mouse olfactory system (Yoshihara et al., 2005), and loss of sonic hedgehog signaling molecule (*SHH*, |log_2_FC| 1.02, *q-*value < 0.05) or olfactomedin 2 (*OLFM2*, |log_2_FC| 1.09, *q-*value < 0.05) impairs olfactory function (Balmer and Lamantia, 2004; Sultana et al., 2014). Moreover, the formation of the olfactory bulb requires fibroblast growth factor (FGF) signaling mediated by the FGF receptor one FGFR1 (|log_2_FC| 0.59, *q-*value < 0.05) (Hébert et al., 2003), and anosmin 1 (*ANOS1*, |log_2_FC| 1.68, *q-*value < 0.05), which is essential for blood vessel formation in the olfactory bulb (Matsushima et al., 2020). So, modifications in the expression of these and other genes might result in changes of smell perception and/or of behavioral responses associated with it as a transition to maternity.

More enigmatic are changes in the mRNA expression of a large number of genes known to influence behavioral traits in humans (Supplementary Table S7). For instance, we have observed an upregulation, in the olfactory bulb of pregnant goats, of the dopamine β-hydroxylase gene (*DBH*, |log_2_FC| 0.93, *q-*value < 0.05), which encodes an oxidoreductase that catalyzes the conversion of dopamine to norepinephrine. In mice, the inactivation of the *DBH* gene causes a profound impairment of maternal behavior characterized by the abandonment of pups which die a few days after birth (Thomas and Palmiter, 1997). The dopamine D1 receptor (*DRD1*, |log_2_FC| 1.91, *q-*value < 0.05) and the 5-hydroxytryptamine receptor 2A (*HTR2A*, |log_2_FC| 0.91, *q-*value < 0.05) show an increased expression in the olfactory bulb of pregnant goats. The *DRD1* gene is involved in the regulation of mothering in rats (Mileva-Seitz et al., 2012), while the HTR2A molecule binds to serotonin, a neurotransmitter upregulated during the transition to motherhood with strong effects on maternal care and pup survivability (Angoa-Pérez et al., 2014; Pawluski et al., 2019). However, it is worth emphasizing that interpreting the observed patterns of mRNA expression from a functional perspective is not completely straightforward. For instance, in the olfactory bulb of pregnant goats we have observed an upregulation of the histamine receptor H1 (*HRH1*, |log_2_FC| 1.43, *q-*value < 0.05) and the prion protein (*PRNP*, |log_2_FC| 0.60, *q-*value < 0.05) mRNAs. The inactivation of these genes leads to increased aggression (Tomaz et al., 2014; Naganuma et al., 2017), while in parallel we have detected an upregulation of histamine receptor H3 (*HRH3*, |log_2_FC| 3, *q-*value < 0.05) mRNA, which in zebrafish has the opposite effect (Reichmann et al., 2020). Moreover, we have found that the neurotensin (*NTS*, |log_2_FC| 0.86, *q-*value < 0.05) mRNA is upregulated in the olfactory bulb of pregnant females. Interestingly, the intracerebroventricular injection of neurotensin in mice decreases aggression associated with the maternal protection of the offspring (Gammie et al., 2009).

With regard to olfactory bulb DEGs linked to depression and anxiety, we have found an increased mRNA expression of both antidepressant (Son et al., 2012; Zhou and Zhou, 2014), e.g., lipid droplet biogenesis associated seipin (*BSCL2*, |log_2_FC| 0.64, *q-*value < 0.05), neuritin (*NRN1*, |log_2_FC| 1.21, *q-*value < 0.05) and glycine receptor β (*GLRB*, |log_2_FC| 0.75, *q-*value < 0.05), and pro-depressive or pro-anxiety genes (Knoll and Carlezon, 2010; Su et al., 2018; Liu et al., 2021), e.g., salt inducible kinase 2 (*SIK2*, |log_2_FC| 1.44, *q-*value < 0.05), prodynorphin (*PDYN*, |log_2_FC| 0.95, *q-*value < 0.05) and histamine receptor H1 (*HRH1*, |log_2_FC| 1.41, *q-*value < 0.05) in pregnant goats. Similarly, we have found increased mRNA levels of genes which promote feeding, e.g., neuropeptide Y, *NPY(*|log_2_FC| 1.58, *q-*value < 0.05); C-X-C motif chemokine ligand 14, *CXCL14* (|log_2_FC| 0.99, *q-*value < 0.05); and neural EGFL like 2, *NELL2* (|log_2_FC| 3.13, *q-*value < 0.05); or inhibit it, e.g., G protein-coupled receptor 45, *GPR45* (|log_2_FC| 1.56, *q-*value < 0.05); and acyl-CoA binding domain containing 7, *ACBD7* (|log_2_FC| 0.63, *q-*value < 0.05), as reported in previous studies (Tanegashima et al., 2010; Cui et al., 2016; Jeong et al., 2017; Lanfray and Richard, 2017; Engström Ruud et al., 2020). These findings illustrate the complexity of ascertaining the functional implications of changes in olfactory bulb mRNA expression associated with pregnancy.

There is also a subset of genes with increased expression in the olfactory bulb of pregnant goats which is particularly interesting because they display dual functions related with both olfaction and behavior. Examples of this category are genes encoding hippocalcin (*HPCA*, |log_2_FC| 1.60, *q-*value < 0.05), a mediator of second messenger signaling in the olfactory epithelium which also regulates spatial and associative memory (Mamman et al., 2004; Kobayashi et al., 2005), and the brain derived neurotrophic factor (*BDNF*, |log_2_FC| 1.78, *q-*value < 0.05), which is important for olfactory bulb neurogenesis, learning and memory (Yuan, 2008; Miranda et al., 2019).

The results of the differential expression analysis should be interpreted with caution because sample size is small. Despite this caveat, we consider that the disproportionate number of DEGs detected in the olfactory bulb when compared to other brain regions is a robust result. Importantly, this anatomical structure is strongly involved in the development of maternal behavior in mice, i.e., its complete removal eliminates such capacity and, very often, implies that litters are cannibalized by their mothers soon after parturition (Gandelman et al., 1971). Moreover, intrabulbar infusions of an oxytocin antagonist delays maternal behavior in rats, while oxytocin itself has the opposite effect (Yu et al., 1996). In sheep, which are phylogenetically close to goats, disruption of the noradrenergic projections to the olfactory bulb suppresses the establishment of a maternal bond between mother and offspring, probably because such event strongly depends on odor cues (Pissonnier et al., 1985). These results support a critical role of the olfactory bulb in the induction of maternal behavior in mammals, although discrepant findings have also been published (Pissonnier et al., 1985).

It has been proposed that in sheep and goats the increase of estrogen levels in blood during prepartum, combined with the vaginocervical stimulation provoked by the expulsion of the fetus, trigger modifications in a neural network including the main olfactory system, the medial preoptic area and the paraventricular nucleus of the hypothalamus, in order to induce maternal behavior (Poindron et al., 2007). Our transcriptomic data demonstrate that extensive areas of the goat brain manifest changes in their expression profiles much before the peripartum period. These alterations might represent an adaptive response toward preparing the pregnant goat for maternity, and we hypothesize that the olfactory bulb might hold a very relevant role in such process.

## Data Availability

The datasets presented in this study can be found in online repositories. The names of the repository/repositories and accession number(s) can be found below: https://www.ncbi.nlm.nih.gov/, PRJNA808876.

## Abbreviations

C*,* combined score; cDNA, complementary desoxyribonucleic acid; CPM, counts per million; DEGs, differentially expressed genes; FC, fold change; FDR, false discovery rate; GTEx, genotype-tissue expression; KEGG, Kyoto encyclopedia of genes and genomes; mRNA, messenger ribonucleic acid; PC, principal component; PCA, principal component analysis; QC, quality control; RIN, RNA integrity number; RNA, ribonucleic acid.
