# Supplementary material for: A protein-coding gene expression atlas from the brain of pregnant and non-pregnant goats
Source: Front Genet. 2023 Jul 14;14:1114749. doi: 10.3389/fgene.2023.1114749 (PMC10382233; doi:10.3389/fgene.2023.1114749)
Supplement: Supplementary file 10 [file DataSheet1.docx]

Supplementary Material

A protein-coding gene expression atlas from the brain of pregnant and non-pregnant goats

**María Gracia Luigi-Sierra, Dailu Guan, Manel López-Béjar, Encarna Casas, Sergi Olvera-Maneu, Jaume Gardela, María Jesús Palomo, Uchebuchi Ike Osuagwuh, Uchechi Linda Ohaneje, Emilio Mármol-Sánchez, Marcel Amills***

*** Correspondence:** Marcel Amills: [marcel.amills@uab.cat](about:blank)

# Supplementary Figures


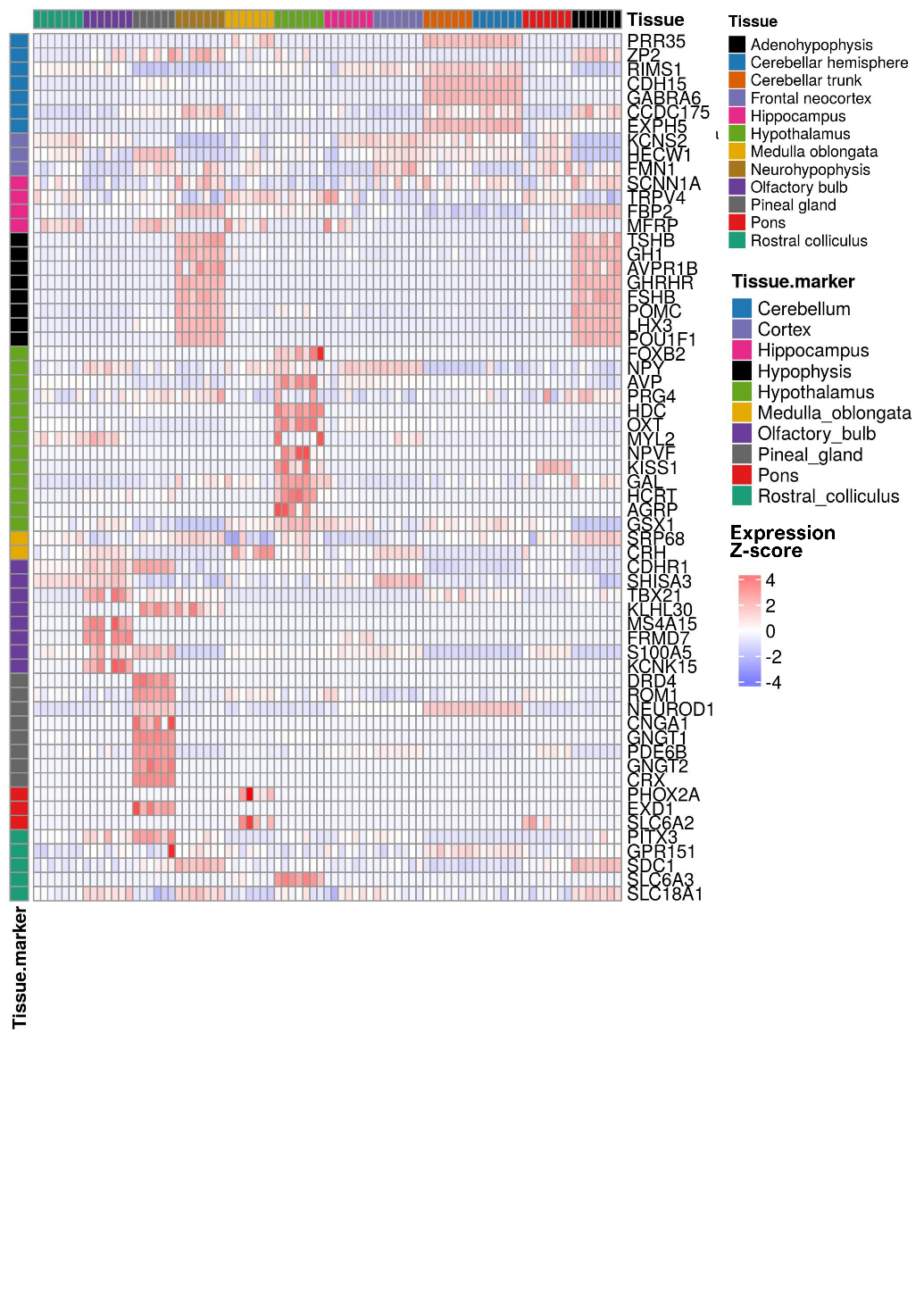


**Supplementary Figure 1.** Heatmap based on the mRNA levels of 61 genes expressed in the goat brain and selected by their high tissue-specificity in a set of 12 human tissues (https://www.proteinatlas.org/, version 22). It can be seen that in goats most of these genes also show highly tissue-specific profiles of mRNA expression for samples collected for each one of the 12 brain regions investigated in the current work. Expression values for each gene (row) are normalized across all samples (columns) by computing the corresponding Z-score.


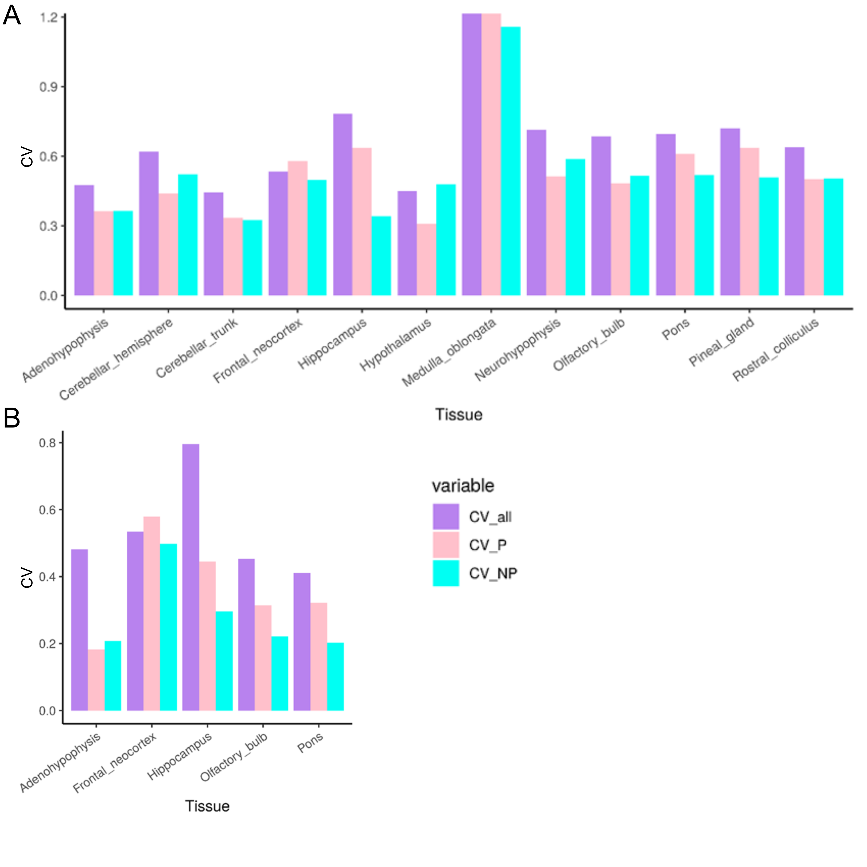


**Supplementary Figure 2.** Coefficient of variation of the gene expression of **(A)** 17,054 Genes expressed in 12 goat brain regions; and **(B)** Genes that are differentially expressed in five tissues (Adenohypophysis, frontal neocortex, hippocampus, olfactory bulb and pons) when comparing pregnant (P) and non-pregnant goats (NP). It can be seen that the variability of mRNA expression within each one of these two P and NP groups is quite limited when contrasted with the gene expression variation observed when samples from all seven goats are pooled together in a single group (All).


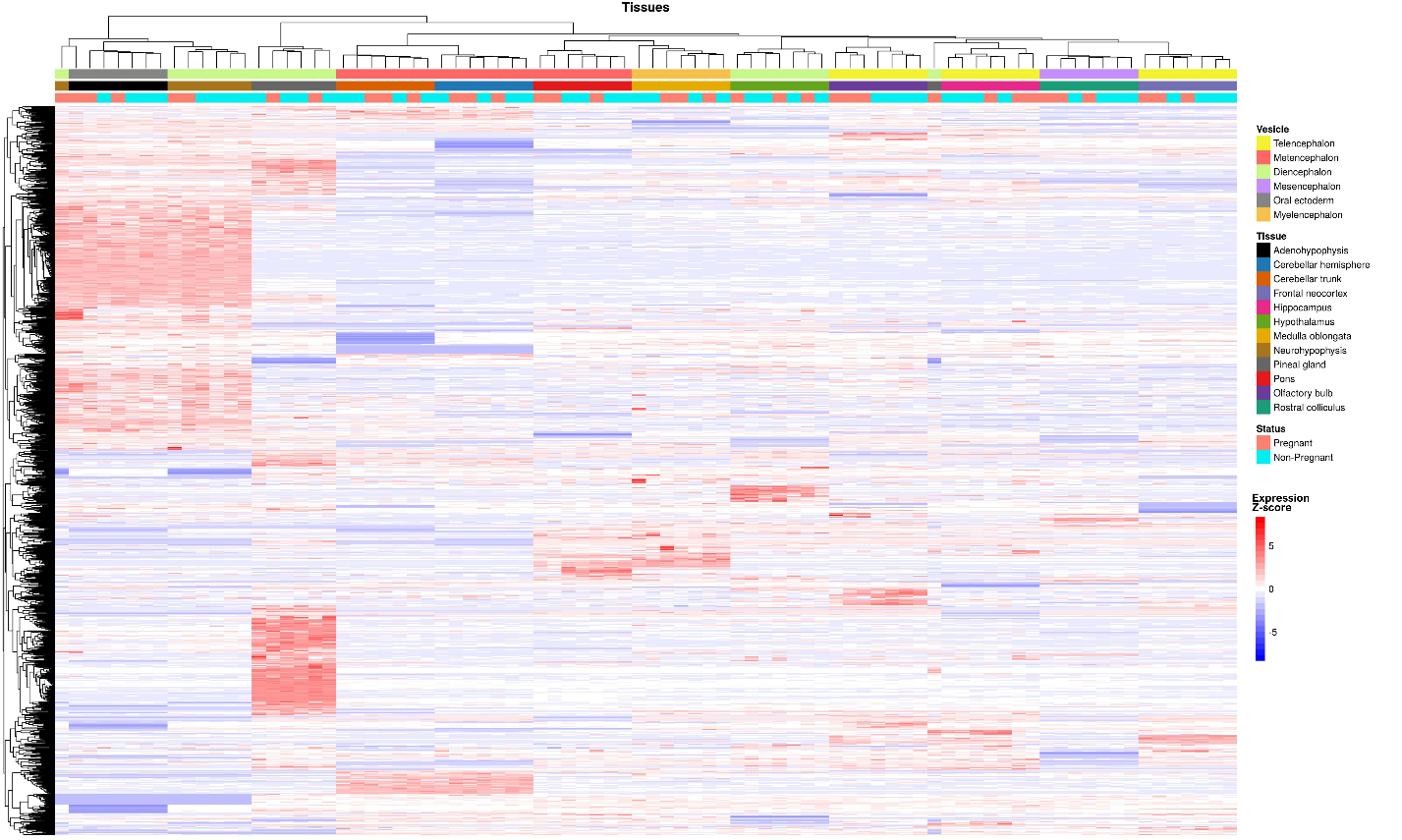


**Supplementary Figure 3.** Heatmap based on the levels of expression of tissue-specific genes (𝜏 ≥ 0.85) identified for each goat brain tissue. Expression values for each gene (row) are normalized across all samples (columns) by computing the corresponding Z-score.


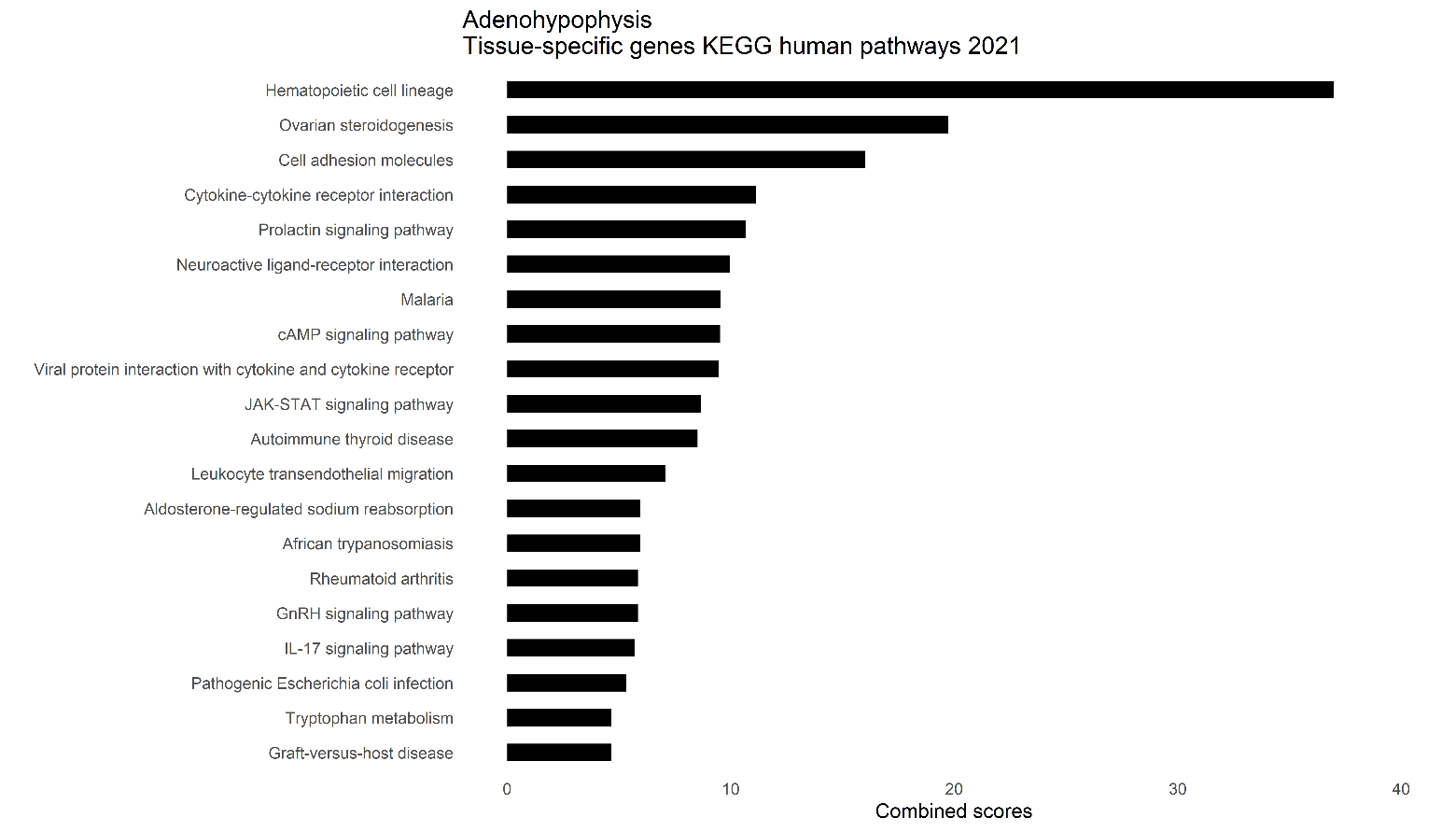


**Supplementary Figure 4.** Top 20 pathways enriched in tissue-specific genes expressed in the adenohypophysis of Murciano-Granadina goats. Combined scores generated in the enrichment analysis are shown in the *x*-axis; while pathway denominations are indicated in the *y*-axis.

**
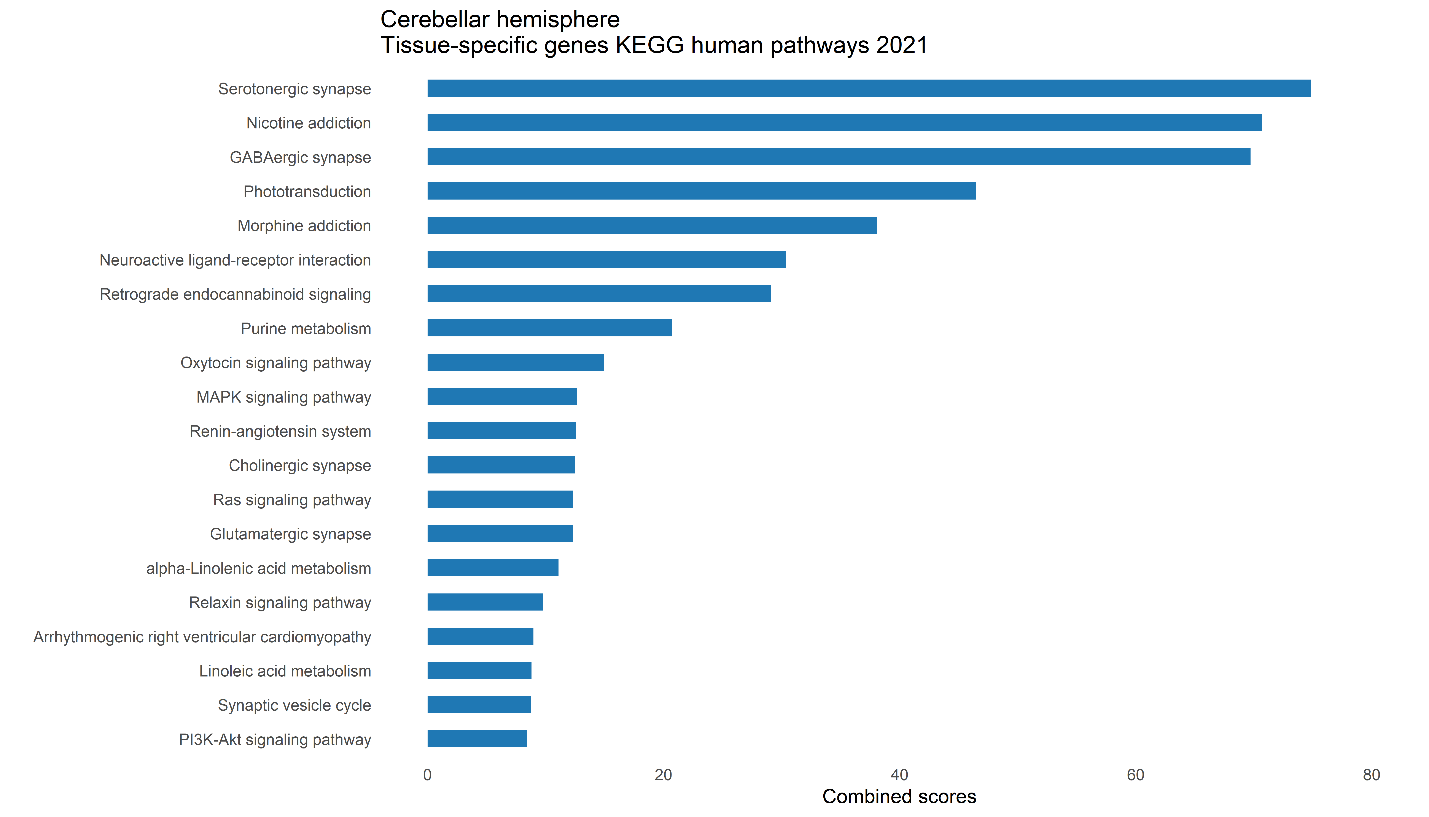
Supplementary Figure 5.** Top 20 pathways enriched in tissue-specific genes expressed in the cerebellar hemisphere of Murciano-Granadina goats. Combined scores generated in the enrichment analysis are shown in the *x*-axis; while pathway denominations are indicated in the *y*-axis.

**Supplementary Figure 3.** Top 20 pathways enriched in tissue-specific genes expressed in the cerebellar hemisphere of Murciano-Granadina goats. Combined scores generated in the enrichment analysis are shown in the x-axis; while pathway denominations are indicated in the y-axis.

**
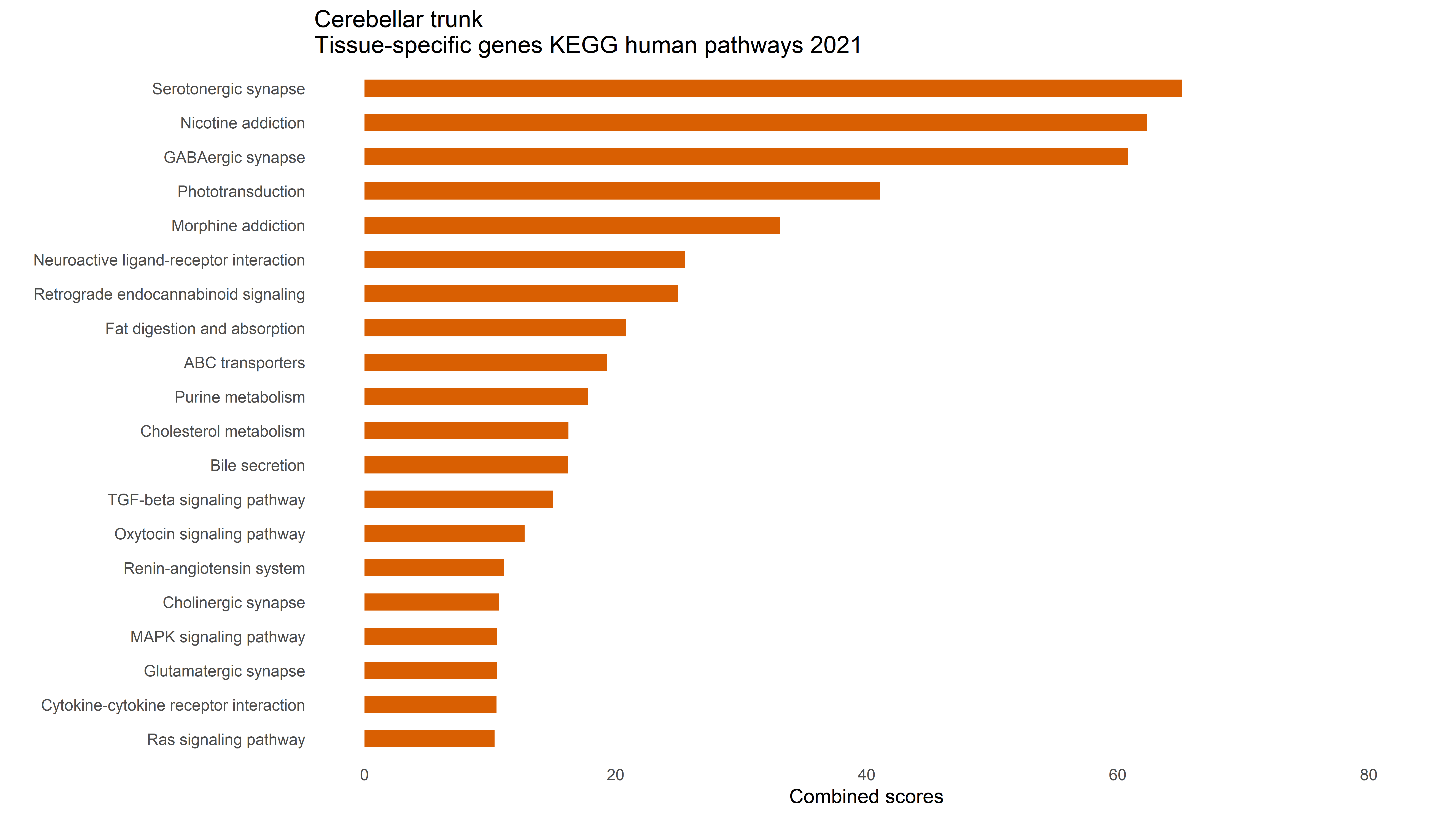
**

**Supplementary Figure 6.** Top 20 pathways enriched in tissue-specific genes expressed in the cerebellar trunk of Murciano-Granadina goats. Combined scores generated in the enrichment analysis are shown in the *x*-axis; while pathway denominations are indicated in the *y*-axis.

**
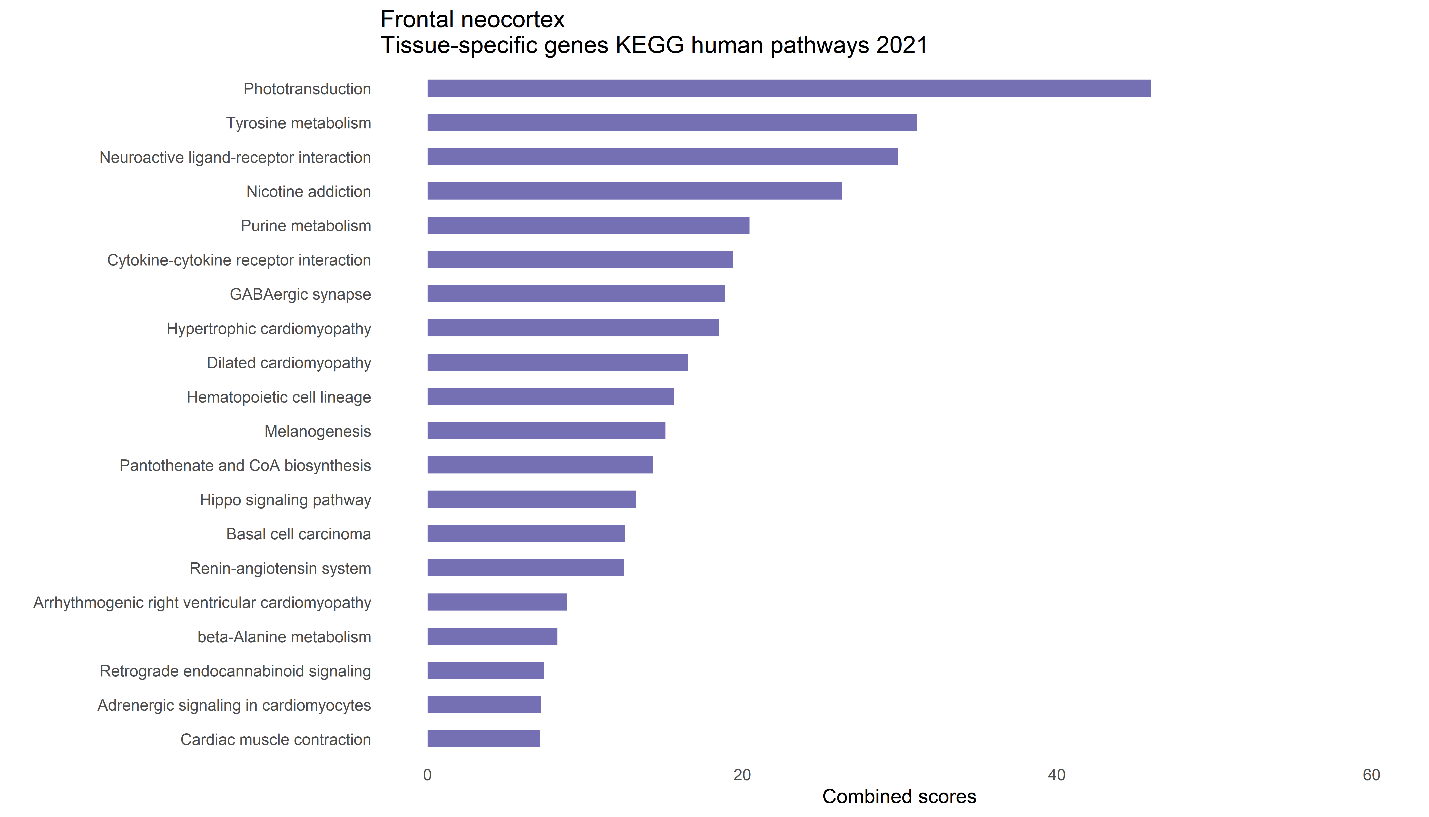
**

**Supplementary Figure 7.**  Top 20 pathways enriched in tissue-specific genes expressed in the frontal neocortex of Murciano-Granadina goats. Combined scores generated in the enrichment analysis are shown in the *x*-axis; while pathway denominations are indicated in the *y*-axis.


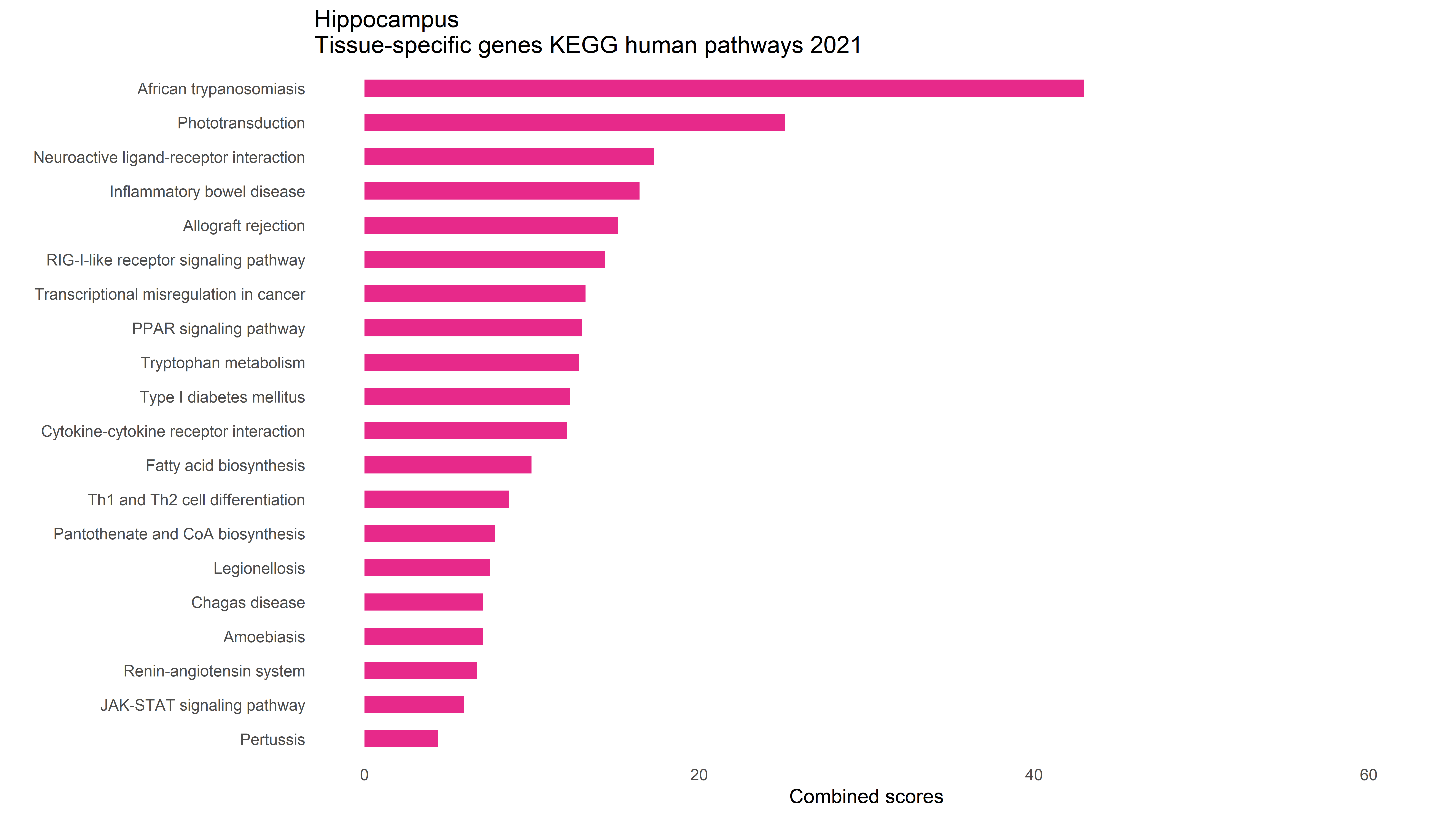


**Supplementary Figure 8.** Top 20 pathways enriched in tissue-specific genes expressed in the hippocampus of Murciano-Granadina goats. Combined scores generated in the enrichment analysis are shown in the *x*-axis; while pathway denominations are indicated in the *y*-axis.


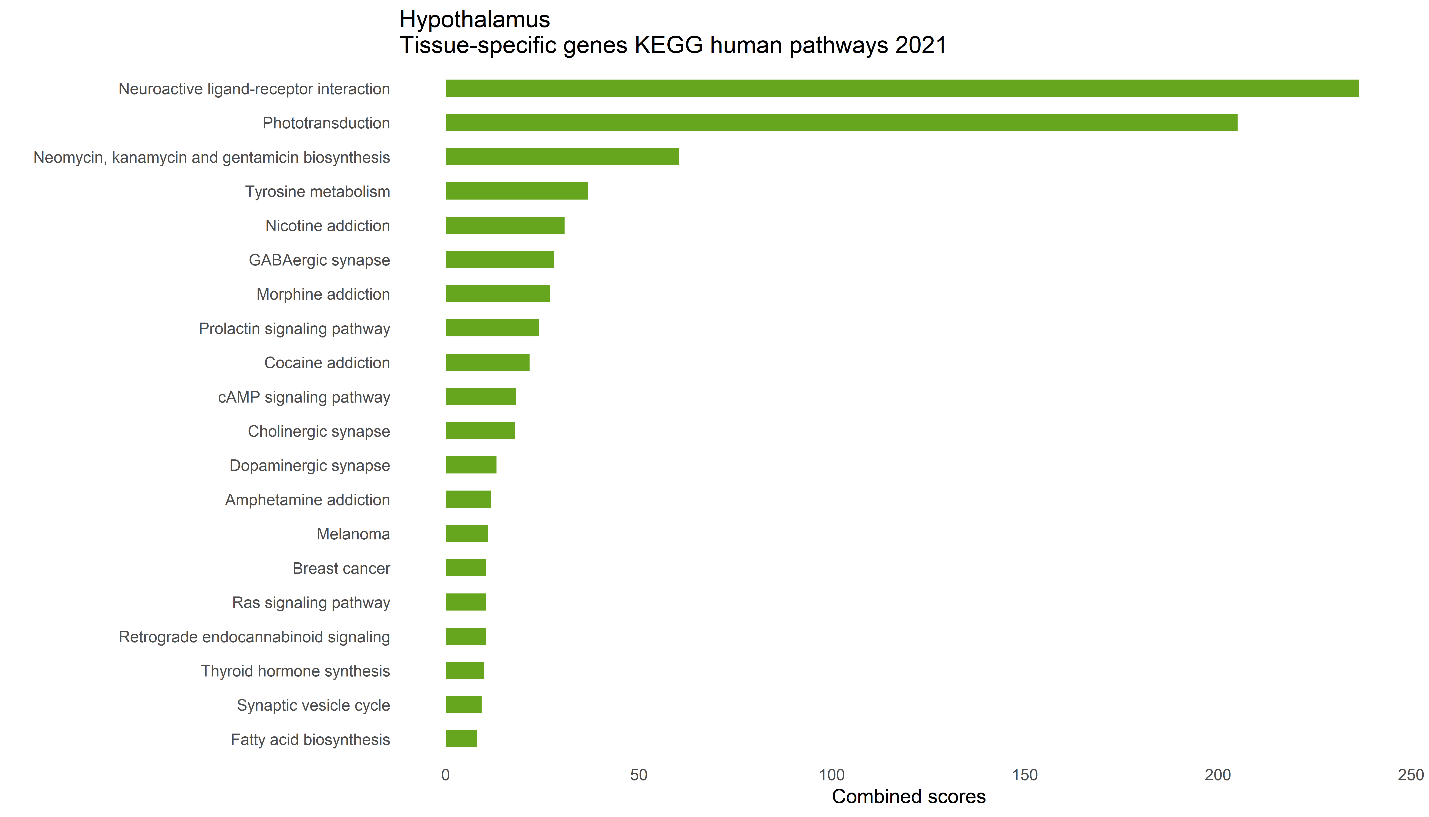


**Supplementary Figure 9.** Top 20 pathways enriched in tissue-specific genes expressed in the hypothalamus of Murciano-Granadina goats. Combined scores generated in the enrichment analysis are shown in the *x*-axis; while pathway denominations are indicated in the *y*-axis.


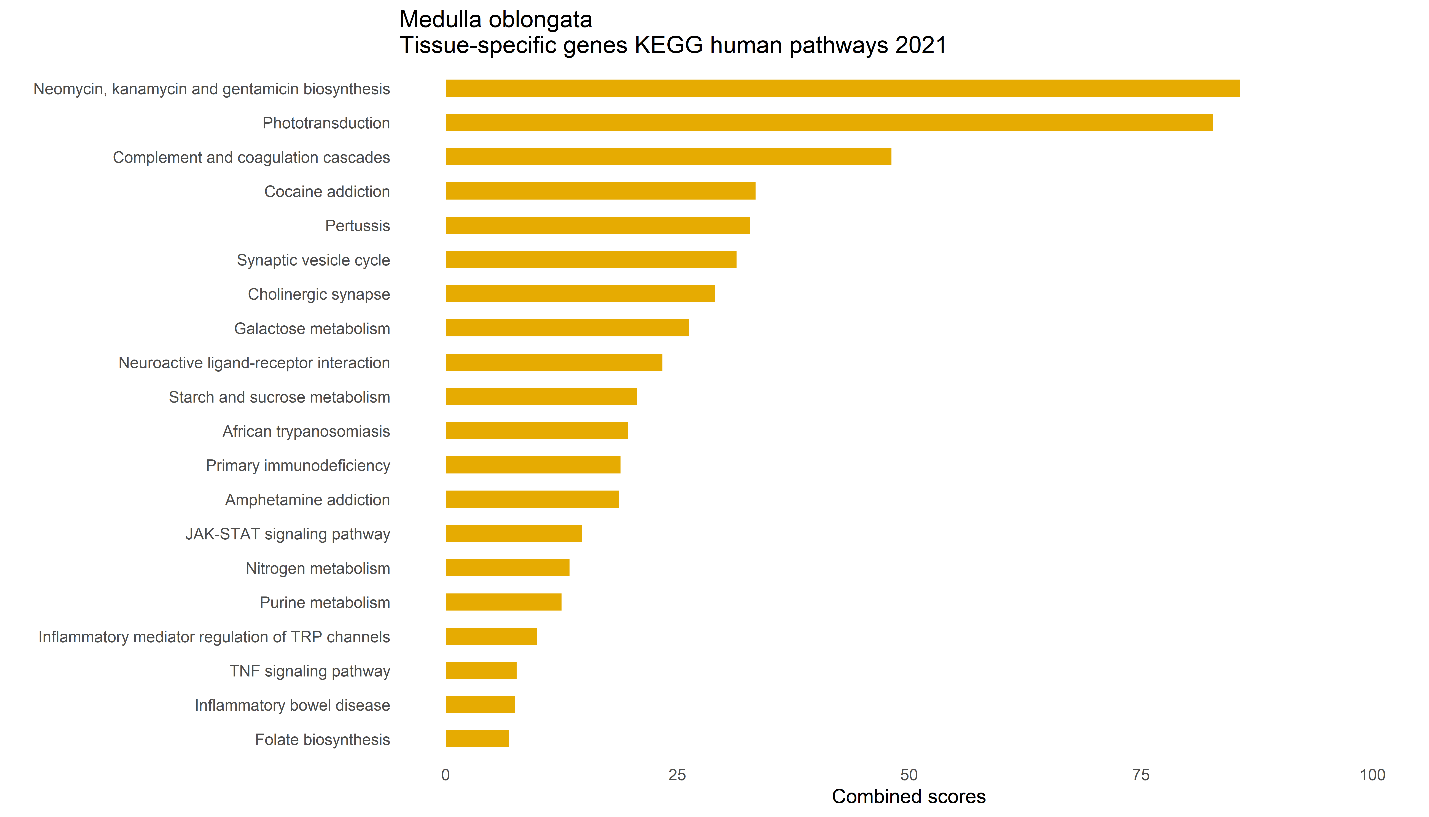


**Supplementary Figure 10.** Top 20 pathways enriched in tissue-specific genes expressed in the medulla oblongata of Murciano-Granadina goats. Combined scores generated in the enrichment analysis are shown in the *x*-axis; while pathway denominations are indicated in the *y*-axis.


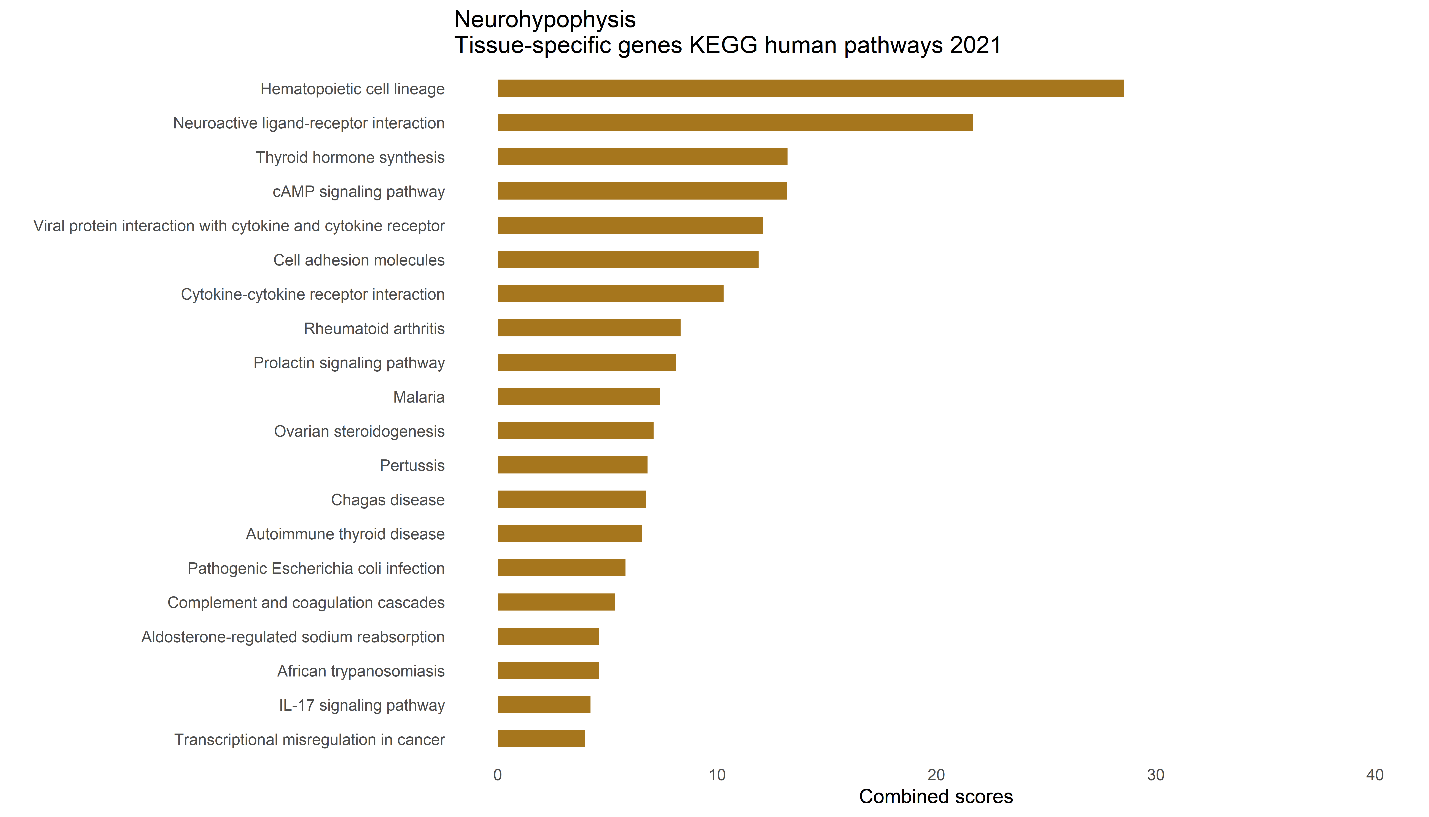


**Supplementary Figure 11.** Top 20 pathways enriched in tissue-specific genes expressed in the neurohypophysis of Murciano-Granadina goats. Combined scores generated in the enrichment analysis are shown in the *x*-axis; while pathway denominations are indicated in the *y*-axis.


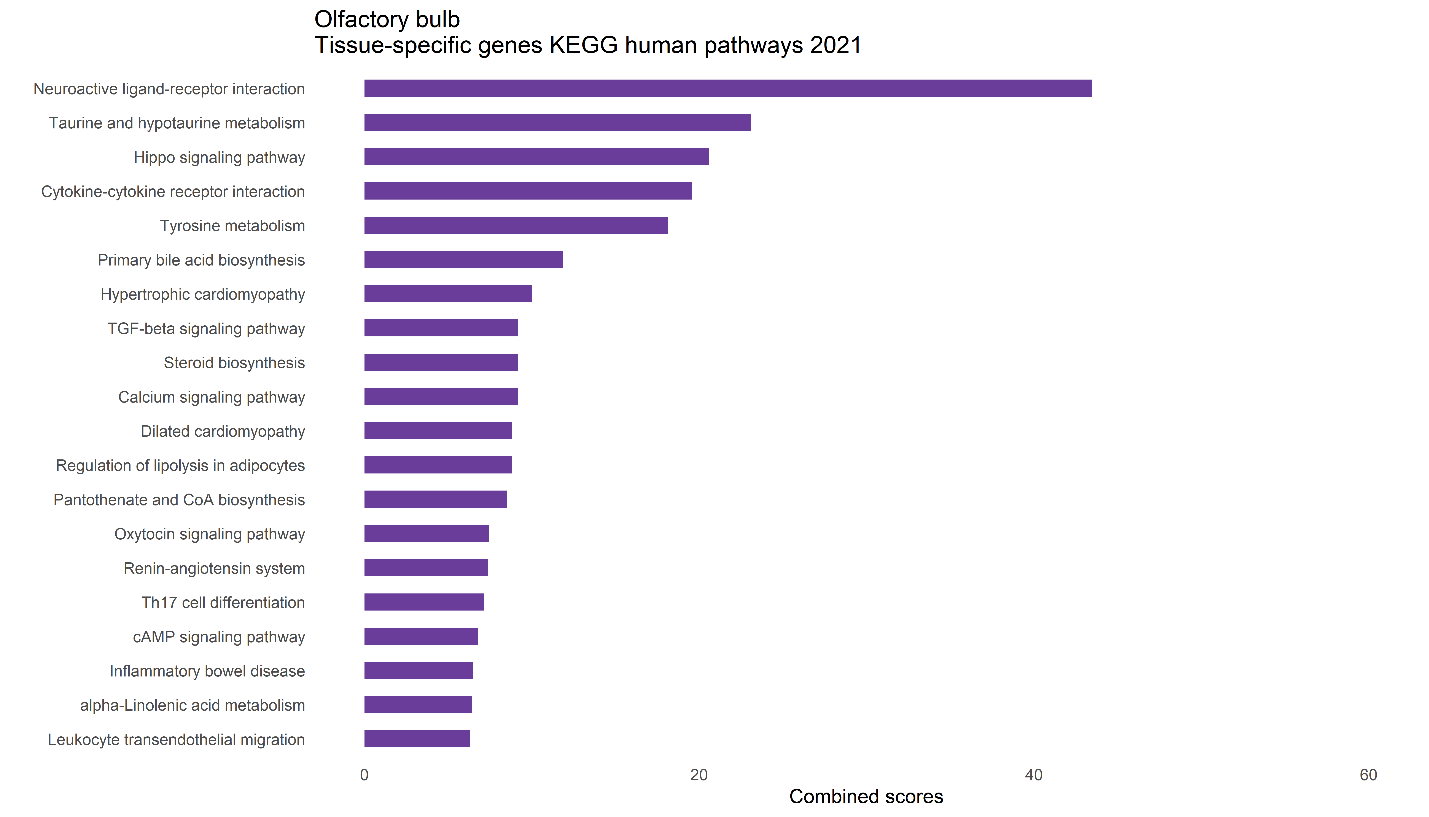


**Supplementary Figure 12.** Top 20 pathways enriched in tissue-specific genes expressed in the olfactory bulb of Murciano-Granadina goats. Combined scores generated in the enrichment analysis are shown in the *x*-axis; while pathway denominations are indicated in the *y*-axis.


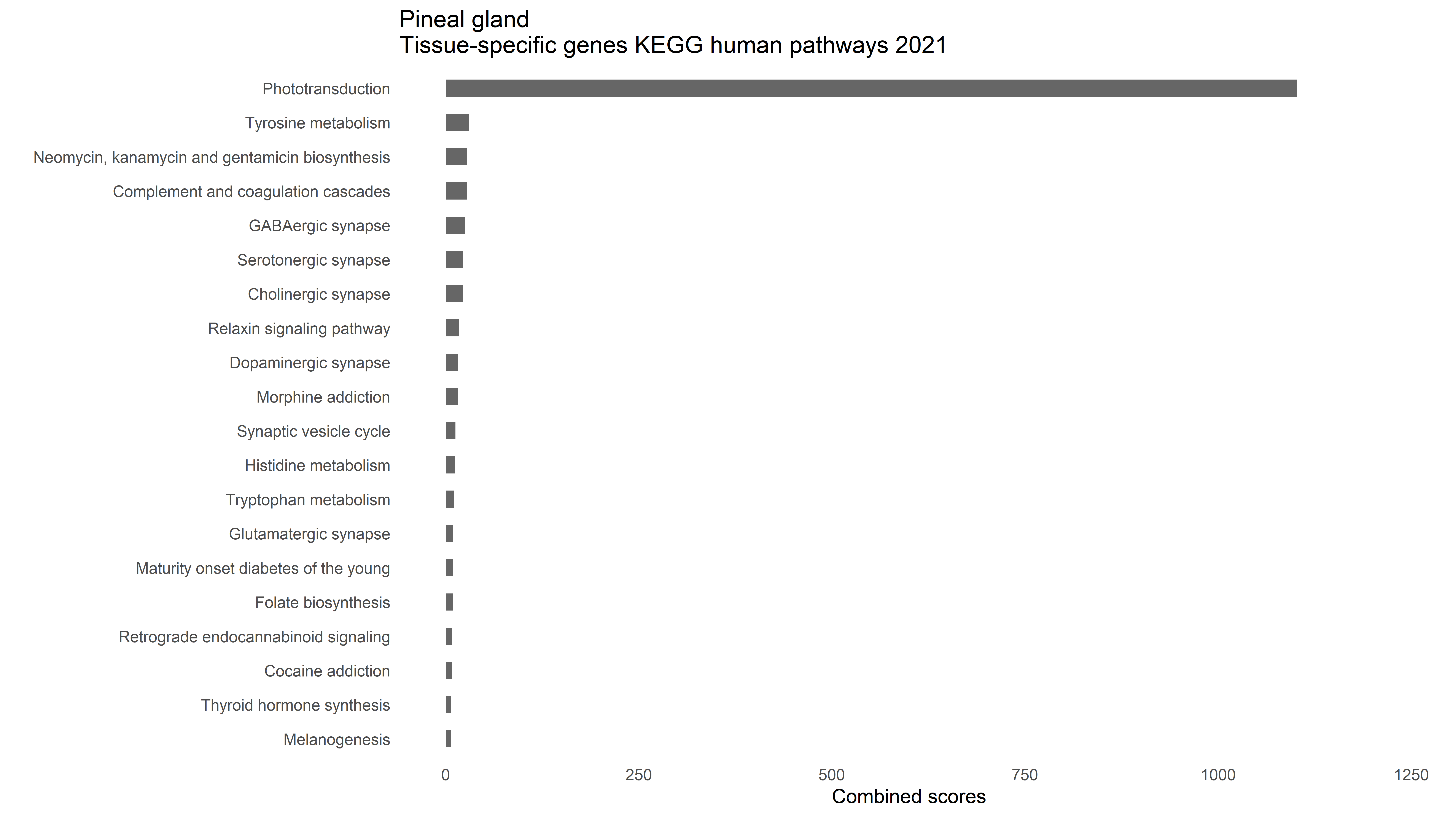


**Supplementary Figure 13.** Top 20 pathways enriched in tissue-specific genes expressed in the pineal gland of Murciano-Granadina goats. Combined scores generated in the enrichment analysis are shown in the *x*-axis; while pathway denominations are indicated in the *y*-axis.


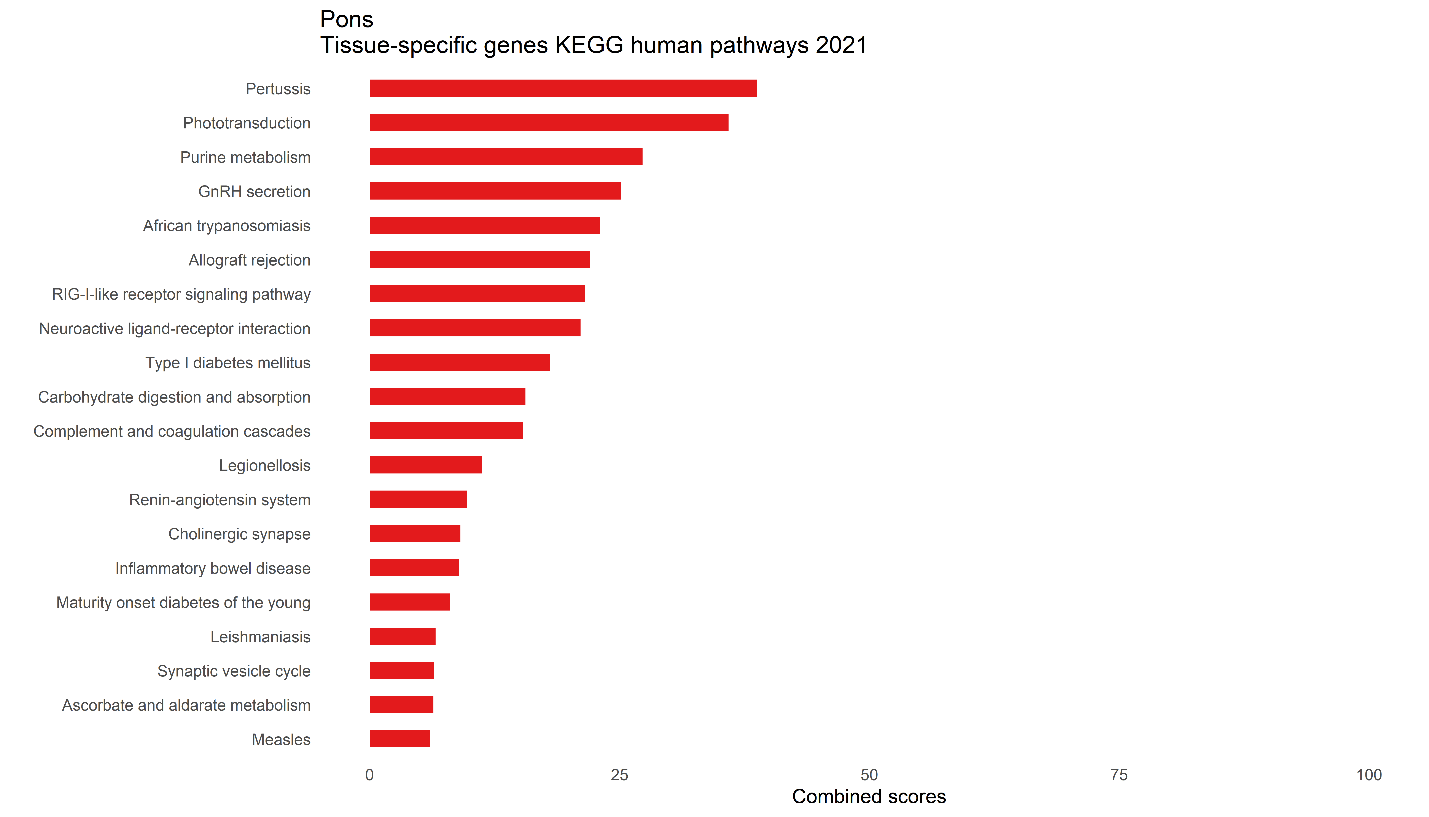


**Supplementary Figure 14.** Top 20 pathways enriched in tissue-specific genes expressed in the pons of Murciano-Granadina goats. Combined scores generated in the enrichment analysis are shown in the *x*-axis; while pathway denominations are indicated in the *y*-axis.


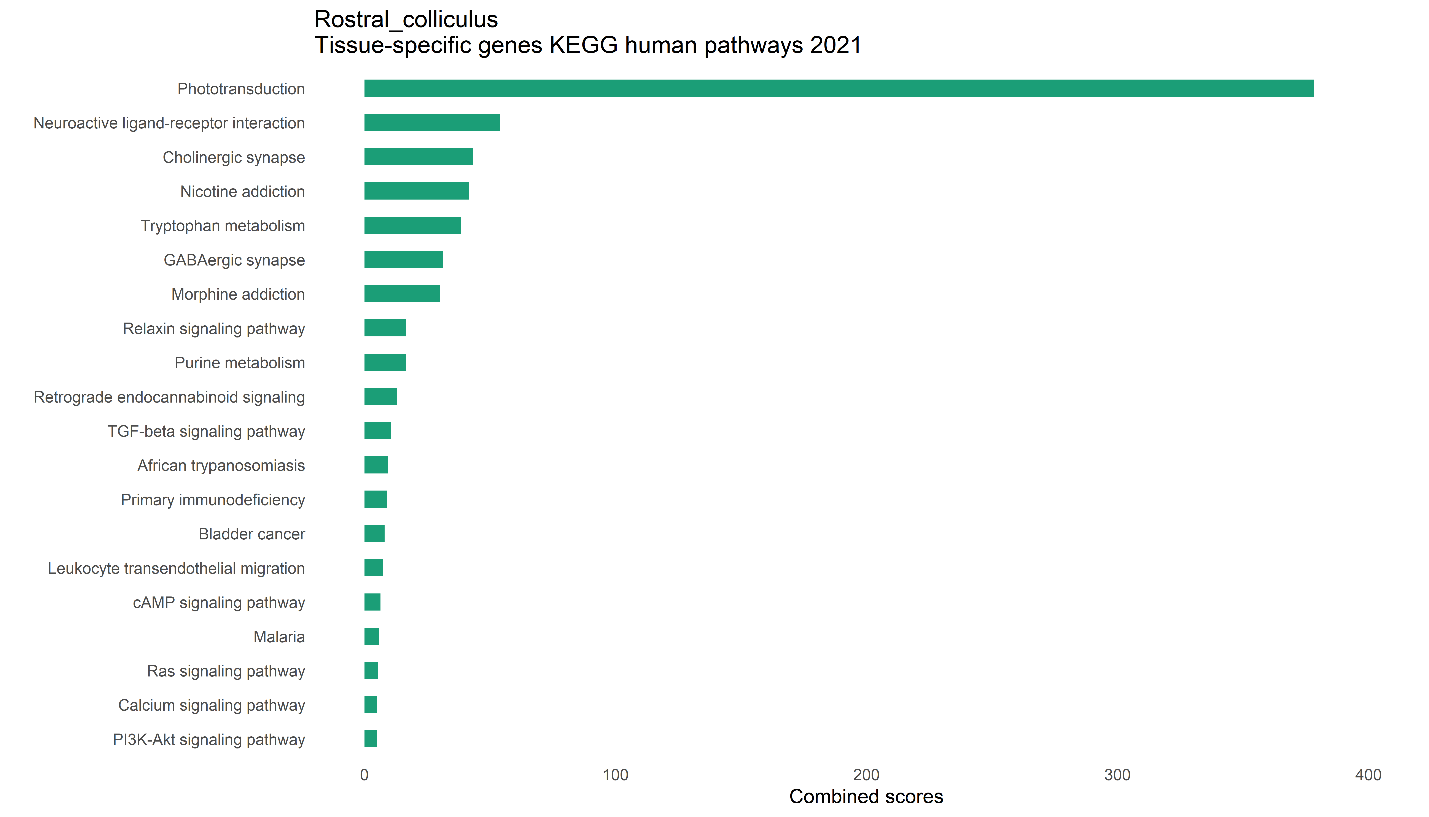


**Supplementary Figure 15.** Top 20 pathways enriched in tissue-specific genes expressed in the rostral colliculus of Murciano-Granadina goats. Combined scores generated in the enrichment analysis are shown in the *x*-axis; while pathway denominations are indicated in the *y*-axis.


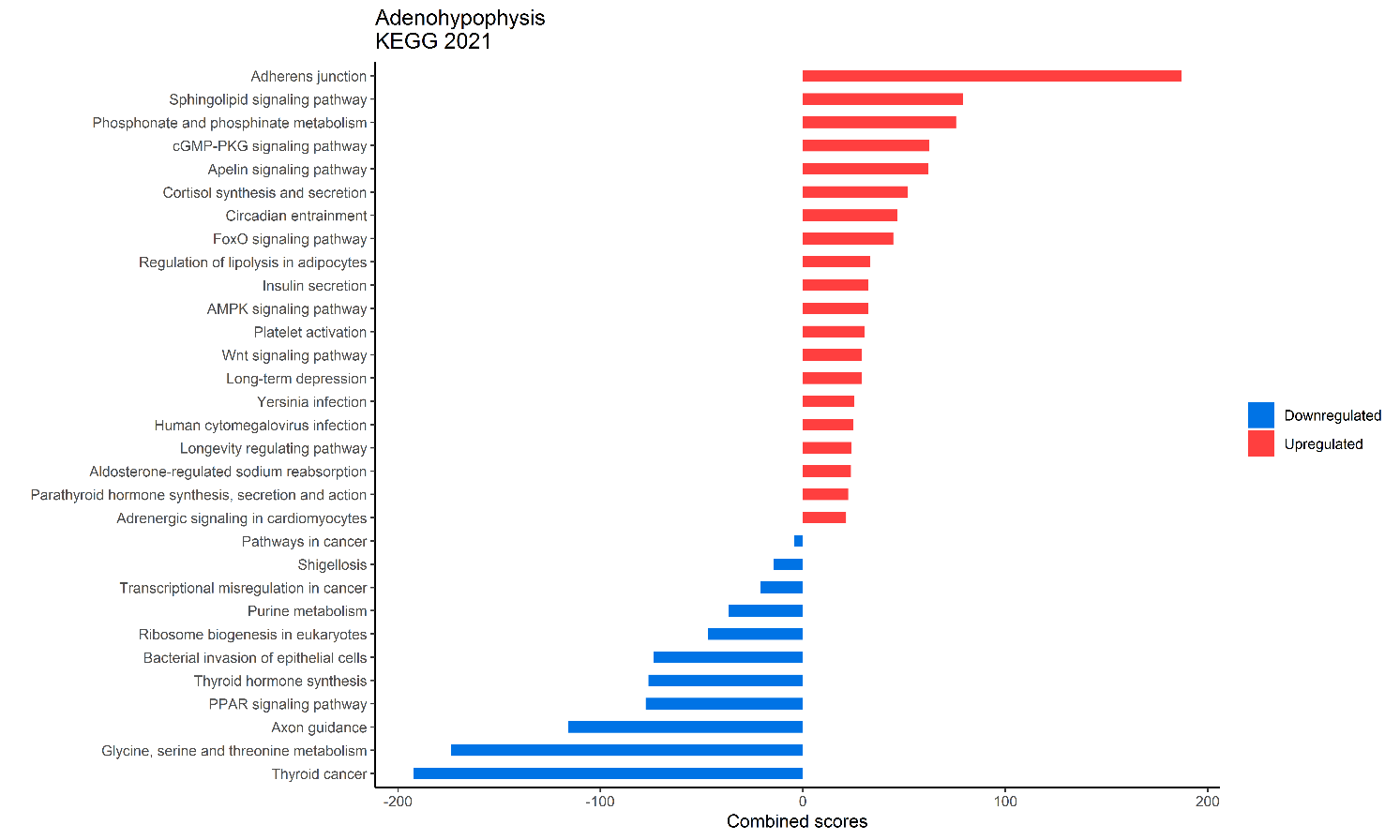


**Supplementary Figure 16.** Pathways enriched in genes differentially expressed in the adenohypophysis of pregnant vs. non-pregnant goats. The *x*-axis displays the combined score calculated from the enrichment analysis, while the *y*-axis indicates the denomination of each pathway. Pathways containing upregulated genes in pregnant goats are displayed in red, while pathways encompassing downregulated genes in pregnant goats are shown in blue.

**Supplementary Figure 14.** Pathways enriched in genes differentially expressed in the adenohypophysis of pregnant vs. non-pregnant goats. The x-axis displays the combined score calculated from the enrichment analysis, while the y-axis indicates the denomination of each pathway. Pathways containing upregulated genes in pregnant goats are displayed in red, while pathways encompassing downregulated genes in pregnant goats are shown in blue.


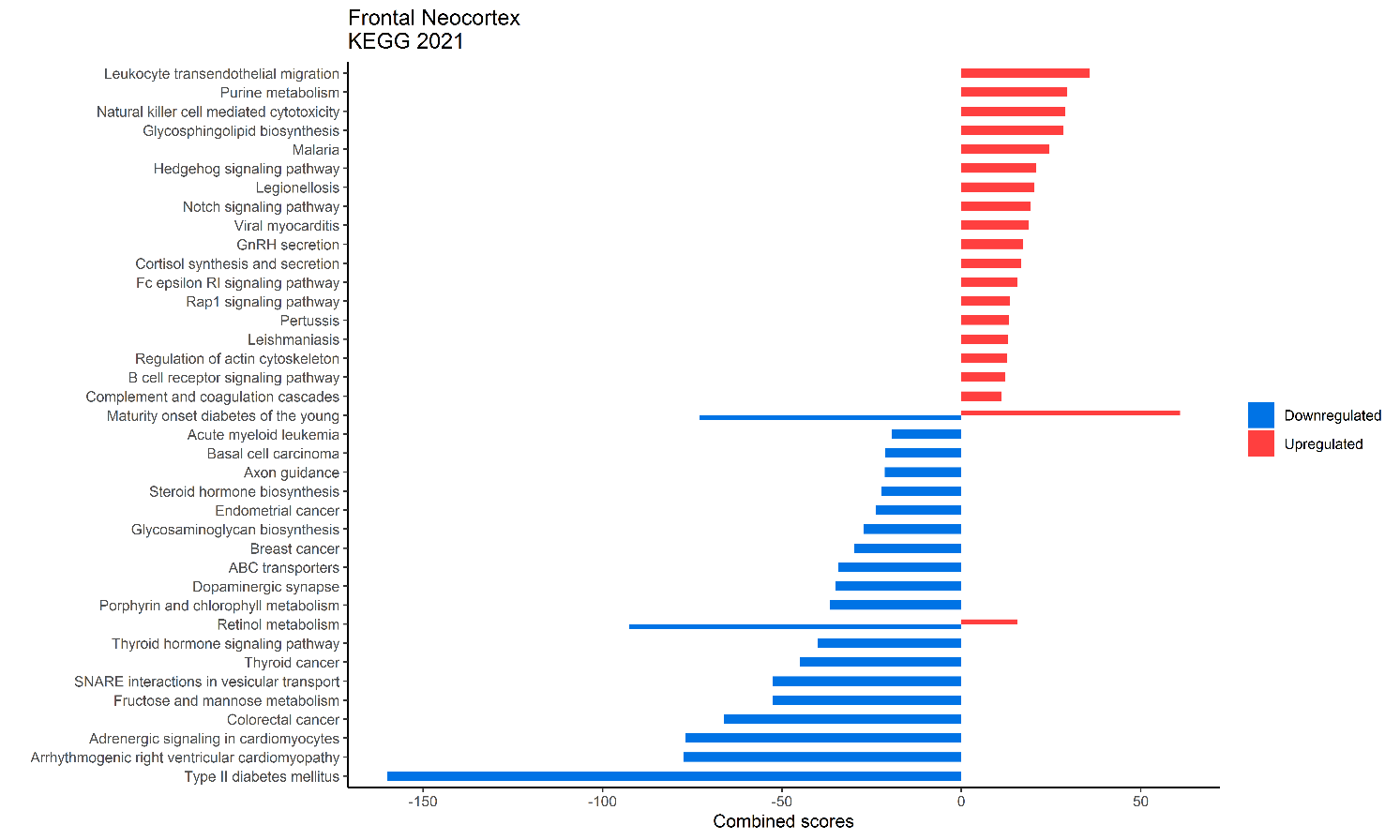


**Supplementary Figure 17.** Pathways enriched in genes differentially expressed in the frontal neocortex of pregnant vs. non-pregnant goats. The *x*-axis displays the combined score calculated from the enrichment analysis, while the *y*-axis indicates the denomination of each pathway

Pathways containing upregulated genes in pregnant goats are displayed in red, while pathways encompassing downregulated genes in pregnant goats are shown in blue.


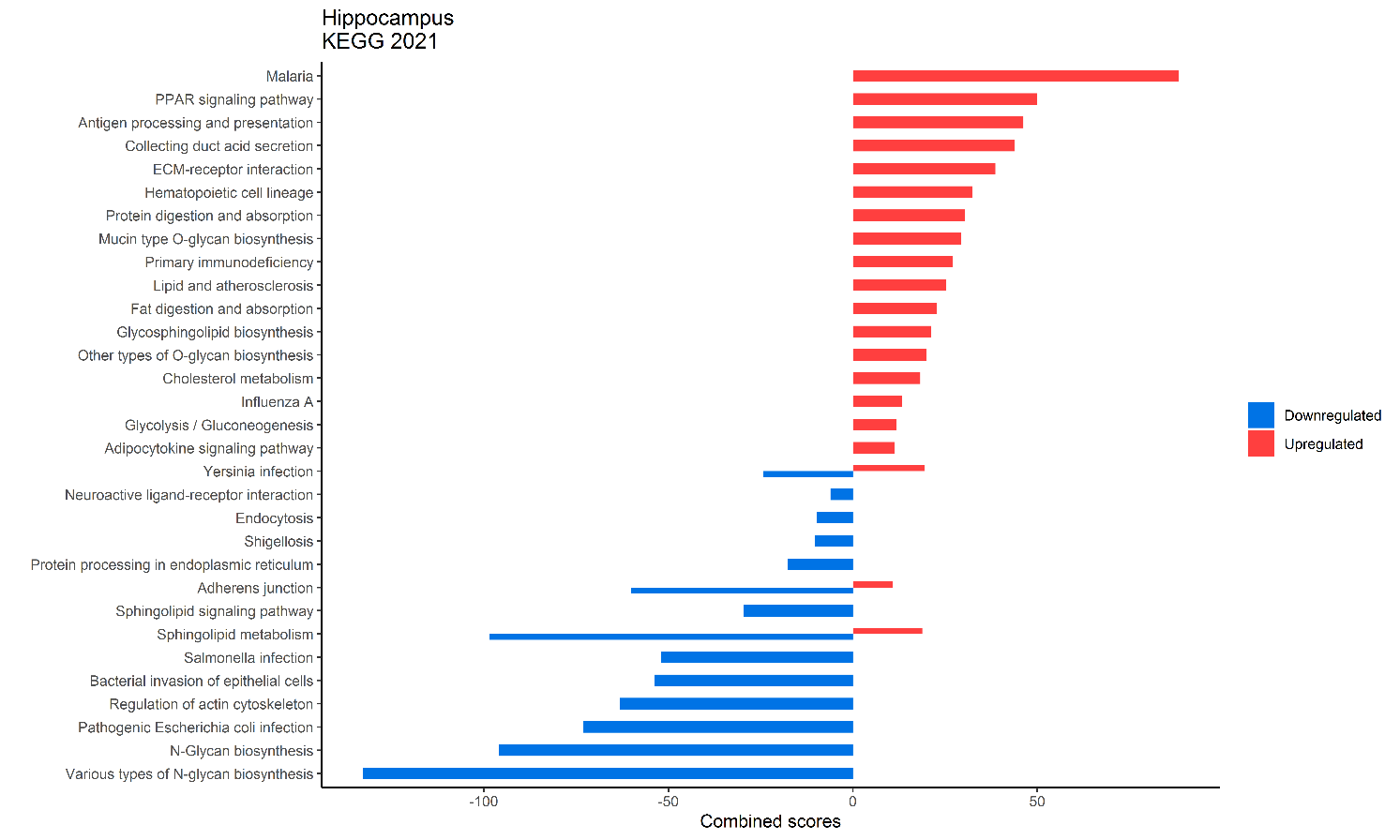


**Supplementary Figure 18.** Pathways enriched in genes differentially expressed in the hippocampus of pregnant vs. non-pregnant goats. The *x*-axis displays the combined score calculated from the enrichment analysis, while the *y*-axis indicates the denomination of each pathway. Pathways containing upregulated genes in pregnant goats are displayed in red, while pathways encompassing downregulated genes in pregnant goats are shown in blue.

Pathways enriched in genes differentially expressed in the hippocampus of pregnant vs. non-pregnant goats. The x-axis displays the combined score calculated from the enrichment analysis, while the y-axis indicates the denomination of each pathway. Pathways containing upregulated genes in pregnant goats are displayed in red, while pathways encompassing downregulated genes in pregnant goats are shown in blue.


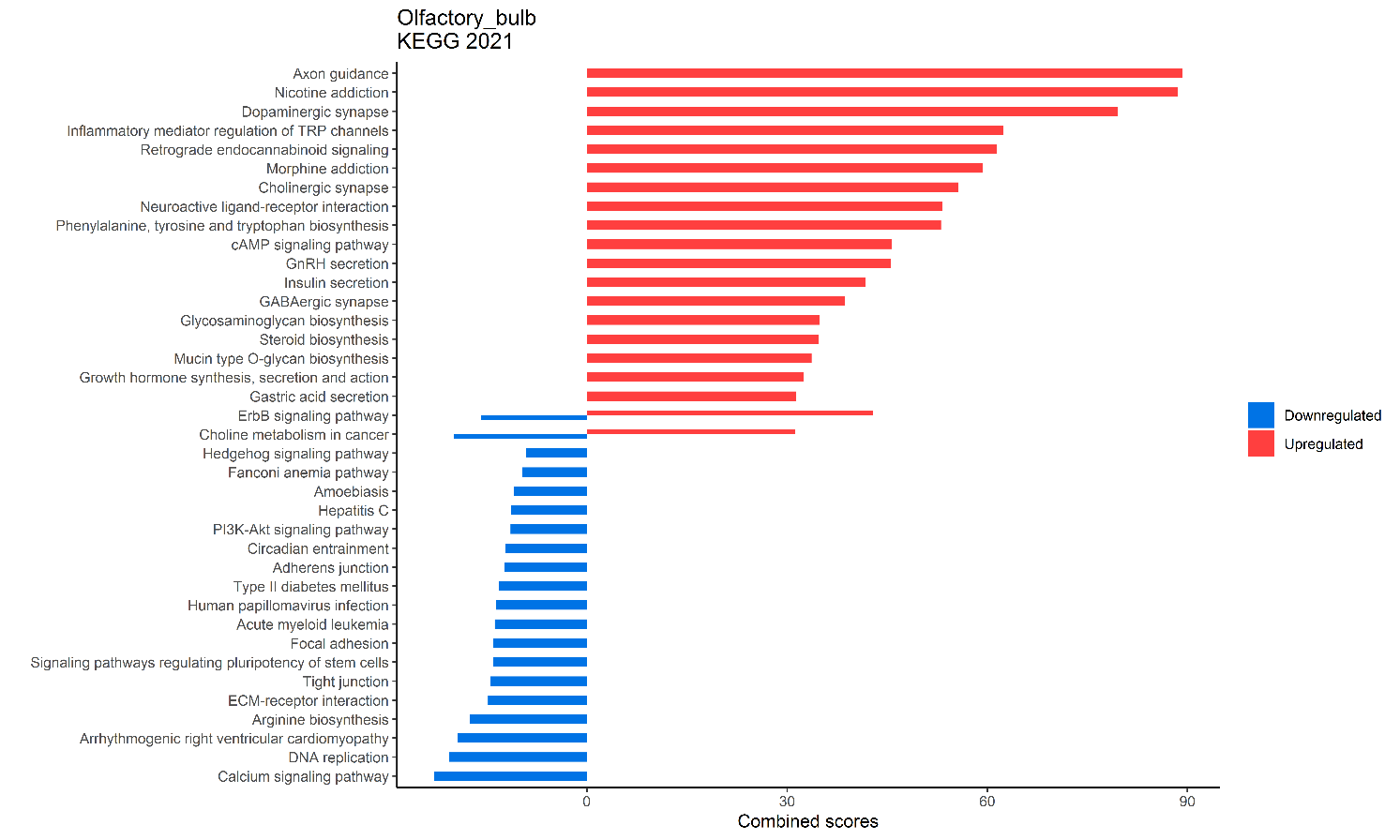


**Supplementary Figure 19.** Pathways enriched in genes differentially expressed in the olfactory bulb of pregnant vs. non-pregnant goats. The *x*-axis displays the combined score calculated from the enrichment analysis, while the *y*-axis indicates the denomination of each pathway. Pathways containing upregulated genes in pregnant goats are displayed in red, while pathways encompassing downregulated genes in pregnant goats are shown in blue.


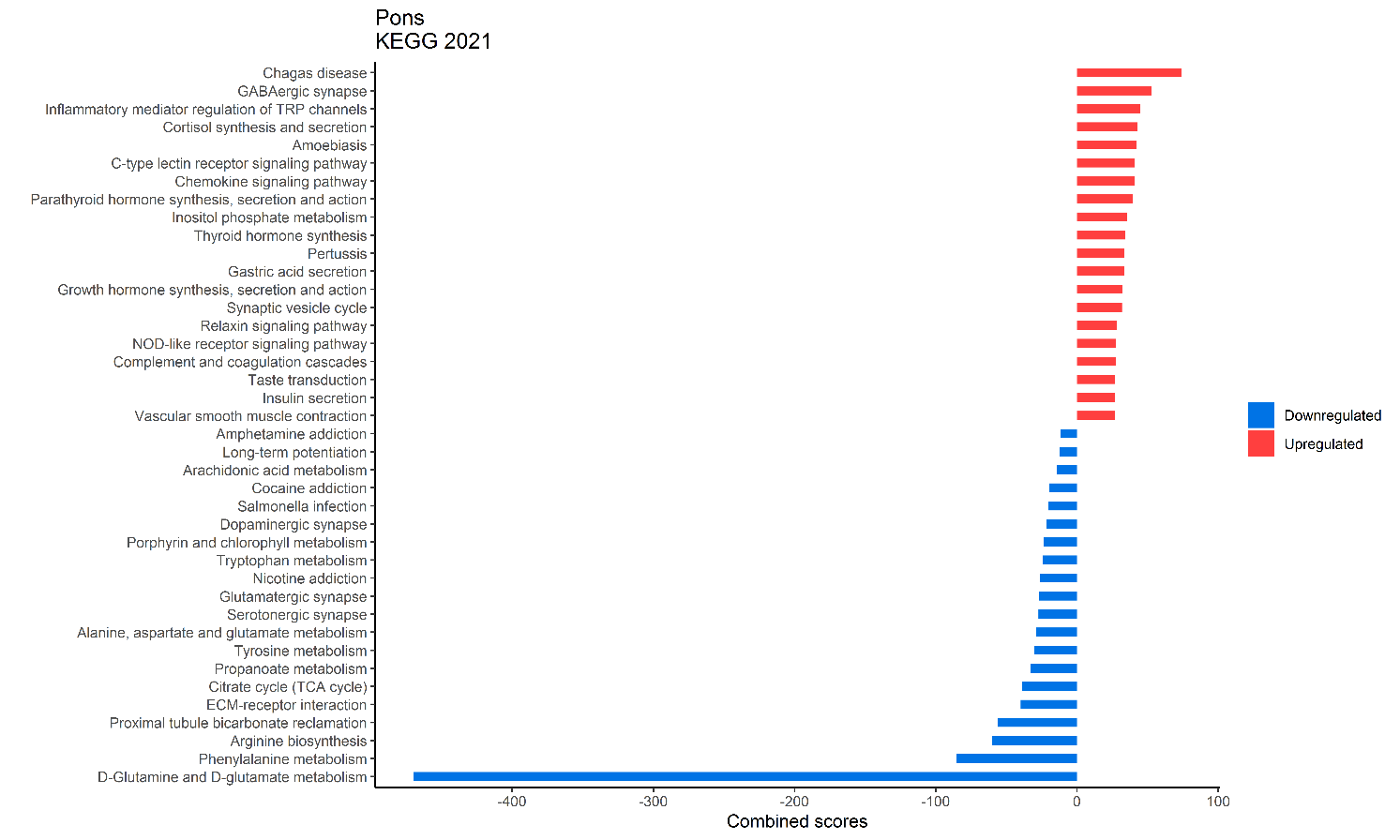


**Supplementary Figure 20.** Pathways enriched in genes differentially expressed in the pons of pregnant goats vs. non-pregnant goats. The *x*-axis displays the combined score calculated from the enrichment analysis, while the *y*-axis indicates the denomination of each pathway. Pathways containing upregulated genes in pregnant goats are displayed in red, while pathways encompassing downregulated genes in pregnant goats are shown in blue.

**
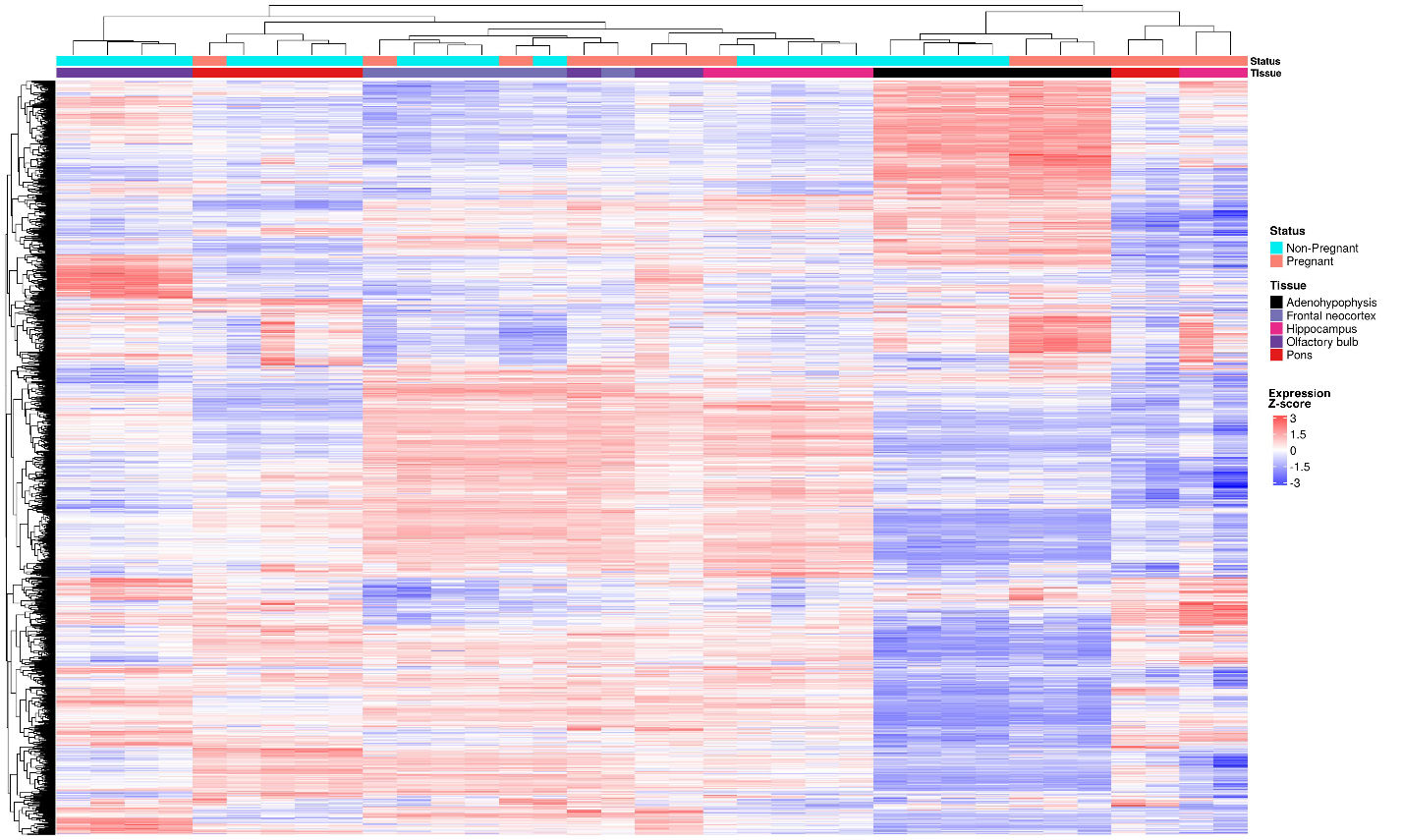
Supplementary Figure 21.** Heatmap based on the levels of expression of differentially expressed genes identified in five tissues moderately or highly affected by pregnancy in goats. Expression values for each gene (row) are normalized across all samples (columns) by computing the corresponding Z-score. It can be seen that the main factor driving differences in gene expression is region-of-sampling rather than pregnancy status.


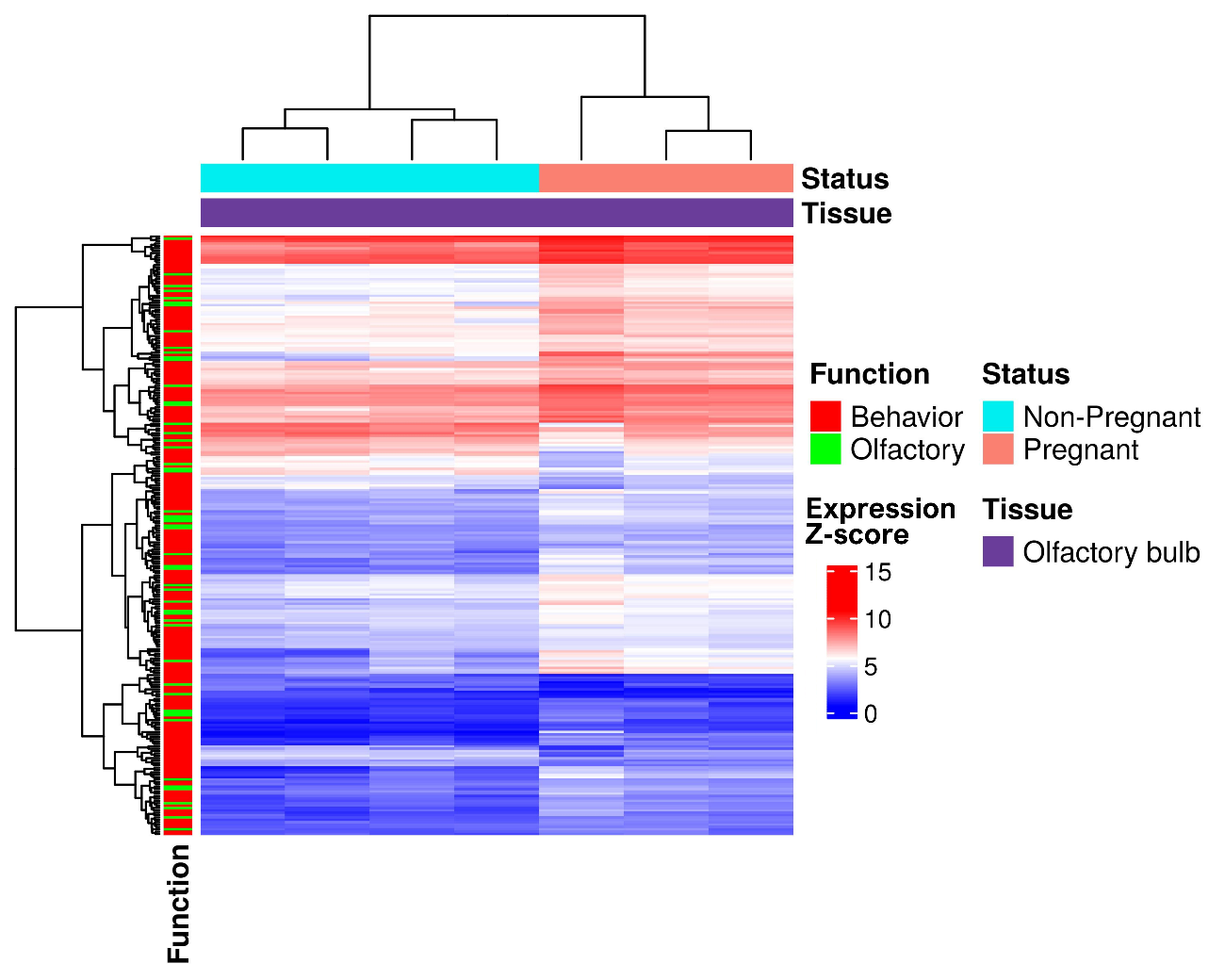


**Supplementary Figure 22.** Heatmap based on the levels of expression of genes differentially expressed in the olfactory bulb of pregnant and non-pregnant goats that are associated with behavioral changes and olfactory functions (see Supplementary Tables 7 and 8 for additional information). Expression values for each gene (row) are normalized across all samples (columns) by computing the corresponding Z-score.
